# Expanding the roles of community health workers to sustain programmes during malaria elimination: a meeting report on operational research in Southeast Asia

**DOI:** 10.1186/s12936-023-04828-4

**Published:** 2024-01-02

**Authors:** Lek Dysoley, James J. Callery, Voeurng Bunreth, Moul Vanna, Chan Davoeung, Yok Sovann, Sles You, Sam Ol, Rupam Tripura, Rusheng Chew, Arjun Chandna, Céline Christiansen-Jucht, Jayme Hughes, Nguon Sokomar, Top Sophornarann, Jeanne Rideout, Tat Veyvath, Oum Sarith, Thaung Puthy, Hay Sothearoth, Sen Sam An, Sazid Ibna Zaman, Lorenz von Seidlein, Lim Vanthy, Preap Sodavuth, Chrun Vannak, Arjen M. Dondorp, Yoel Lubell, Richard J. Maude, Thomas J. Peto, Bipin Adhikari

**Affiliations:** 1 National Centre for Parasitology, Entomology and Malaria Control, Phnom Penh, Cambodia; 2 National Institute for Public Health, Phnom Penh, Cambodia; 3 Mahidol‑Oxford Tropical Medicine Research Unit, Faculty of Tropical Medicine, Mahidol University, Bangkok, Thailand; 4 Centre for Tropical Medicine and Global Health, University of Oxford, Oxford, UK; 5 Provincial Health Department, Battambang, Cambodia; 6 Action for Health Development, Battambang, Cambodia; 7 Provincial Health Department, Pailin, Cambodia; 8 Faculty of Medicine, University of Queensland, Brisbane, Australia; 9 Cambodia Oxford Medical Research Unit, Angkor Hospital for Children, Siem Reap, Cambodia; 10 World Health Organization, Phnom Penh, Cambodia; 11 President’s Malaria Initiative, Phnom Penh, Cambodia; 12 Clinton Health Access Initiative, Phnom Penh, Cambodia; 13 Cambodia Malaria Elimination Project 2, Phnom Penh, Cambodia; 14 University Research Company Ltd, Phnom Penh, Cambodia; 15 United States Agency for International Development, Phnom Penh, Cambodia; 16 United Nations Office for Project Services, Phnom Penh, Cambodia; 17 The Open University, Milton Keynes, UK

**Keywords:** Malaria, Community health workers, Village malaria workers, Roles, Malaria elimination

## Abstract

In Southeast Asia malaria elimination is targeted by 2030. Cambodia aims to achieve this by 2025, driven in large part by the urgent need to control the spread of artemisinin-resistant falciparum malaria infections. Rapid elimination depends on sustaining early access to diagnosis and effective treatment. In much of Cambodia, rapid elimination will rely on a village malaria worker (VMW) network. Yet as malaria declines and is no longer a common cause of febrile illness, VMWs may become less popular with febrile patients, as VMWs do not diagnose or treat other conditions at present. There is a risk that VMWs become inactive and malaria rebounds before the complete interruption of transmission is achieved.

During 2021–23 a large-scale operational research study was conducted in western Cambodia to explore how a VMW network could be sustained by including health activities that cover non-malarial illnesses to encourage febrile patients to continue to attend. 105 VMWs received new rapid diagnostic tests (including dengue antigen–antibody and combined malaria/C-reactive protein tests), were trained in electronic data collection, and attended health education packages on hygiene and sanitation, disease surveillance and first aid, management of mild illness, and vaccination and antenatal care.

In August 2023 the National Malaria Control Programme of Cambodia convened a stakeholder meeting in Battambang, Cambodia. Findings from the study were reviewed in the context of current malaria elimination strategies. The discussions informed policy options to sustain the relevance of the VMW network in Cambodia, and the potential for its integration with other health worker networks. This expansion could ensure VMWs remain active and relevant until malaria elimination is accomplished.

**Supplementary Information:**

The online version contains supplementary material available at 10.1186/s12936-023-04828-4.

## Background

Community health workers (CHWs) are critical to improving the health and well-being of remote rural populations in low-resource settings [1–3]. The roles and contributions of CHWs have evolved over the years based on disease epidemiology, health system priorities, and available resources [4]. Specific cadres of CHWs for malaria in Cambodia are referred to as village malaria workers (VMWs). They are trained to provide malaria diagnosis, treatment, referral, surveillance, and preventive activities [5–7]. The VMW programme in Cambodia was established in 2004 and later expanded to include mobile malaria workers who can provide services to remote and underserved communities [8]. Approximately 3500 VMWs have been deployed in rural areas of Cambodia where malaria is endemic. The VMW programme has been linked to a significant decrease in malaria cases in recent years [9]. This decline was first noticeable among *Plasmodium falciparum* infections and later vivax malaria as well [7, 10, 11]. The programme's success in providing convenient treatment options has been identified as a major factor contributing to this downward trend in malaria incidence [5, 7, 12]. In 2020 alone, VMWs contributed substantially to malaria case management, conducting 73% of malaria tests and detecting 61% of confirmed malaria cases in the country [13].

Over the last two decades, the decline of malaria in Cambodia has been remarkable and the country is now moving towards the stage at which it will certify the elimination of malaria at a sub-national level. The current trends in malaria incidence provide optimism that the National Malaria Control Programme of Cambodia (CNM) will reach its goal of malaria elimination. Cambodia plans to achieve the elimination of all four predominantly human malarias (*P. falciparum, Plasmodium vivax, Plasmodium ovale,* and *Plasmodium malariae*) by 2025, but while navigating the pre-elimination phase, different tools and strategies remain under consideration [14]. Although the reported incidence of falciparum malaria has been reduced to 392 cases in 2022, vivax malaria cases (n = 3566 out of a total of 4021 of all malaria confirmed cases) still remain a stubborn challenge and require a revised strategy for elimination [15]. Approximately 94% of Cambodian malaria cases were caused by *P. vivax* in the first quarter of 2023 [16].

**Fig. 1:**
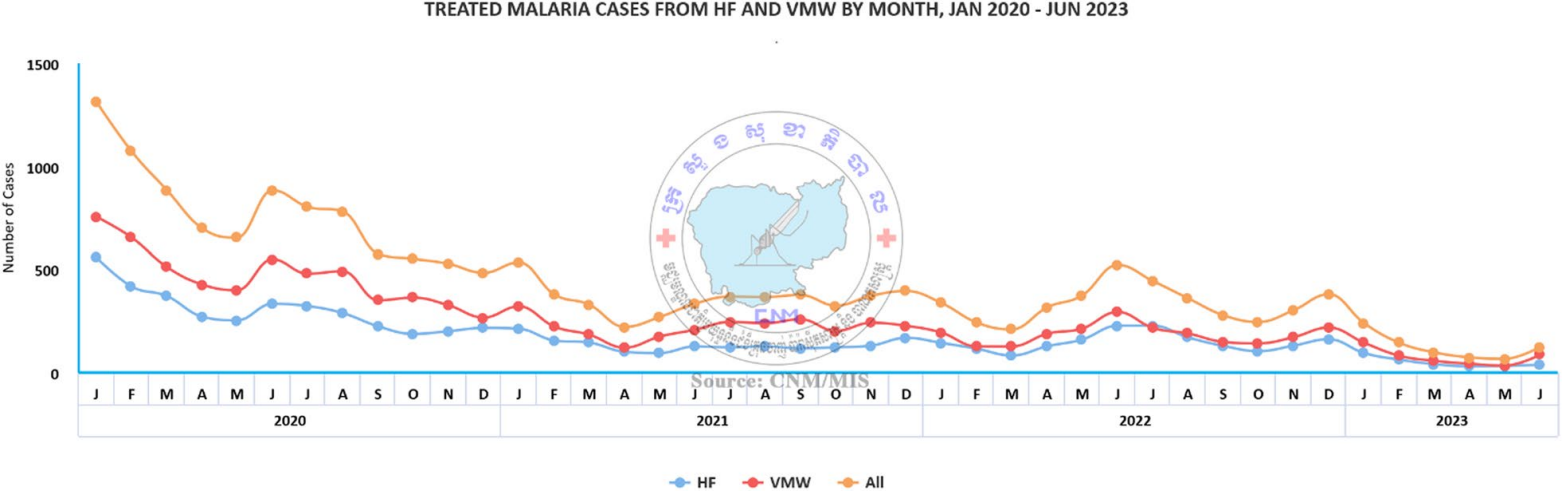
Progressive decline in the number of malaria cases treated by village malaria workers from 2020 to 2023. The figure was derived from CNM’s online malaria information system available online at https://mis.cnm.gov.kh/

Since 2020, the number of malaria cases treated by VMWs has been progressively decreasing in line with the overall decline in malaria transmission (Fig. 1). The decrease in the proportion of fevers caused by malaria represents an opportunity to upskill VMWs to help manage non-malarial febrile illnesses that contribute to significant morbidity and mortality in Cambodia [17]. Expanding their roles helps to preserve these locally available health workers, recognized as the core of the primary healthcare system [5, 18]. In addition to their capability to manage malaria, the local presence of VMWs within a community means they are ideally positioned to provide primary healthcare for various illnesses beyond malaria [19]. CNM has been undertaking discussions around how to expand VMWs’ roles and integrate them into the health system including the uptake of evidence from operational research.

From 2021 to 2023 an operational research project was conducted under the Regional Artemisinin-resistance Initiative 3E (RAI3E) in Cambodia, Lao People’s Democratic Republic and Thailand to assess the potential for VMWs to be sustained through expanding their roles and/or integration into the wider health system. Operational research is a form of embedded health system study that utilizes existing resources (e.g., health structures, service providers, and service recipients) to improve programme operations and thereby deliver more effective care [20–22]. The approaches embedded in operational research can address effective targeting, feasibility, and optimization, bridging the gap between traditional research methods and the more practical side of service and programme implementation [20–23]. Often the findings from operational research are followed by stakeholder meetings where findings are discussed to guide policies.

**Fig. 2:**
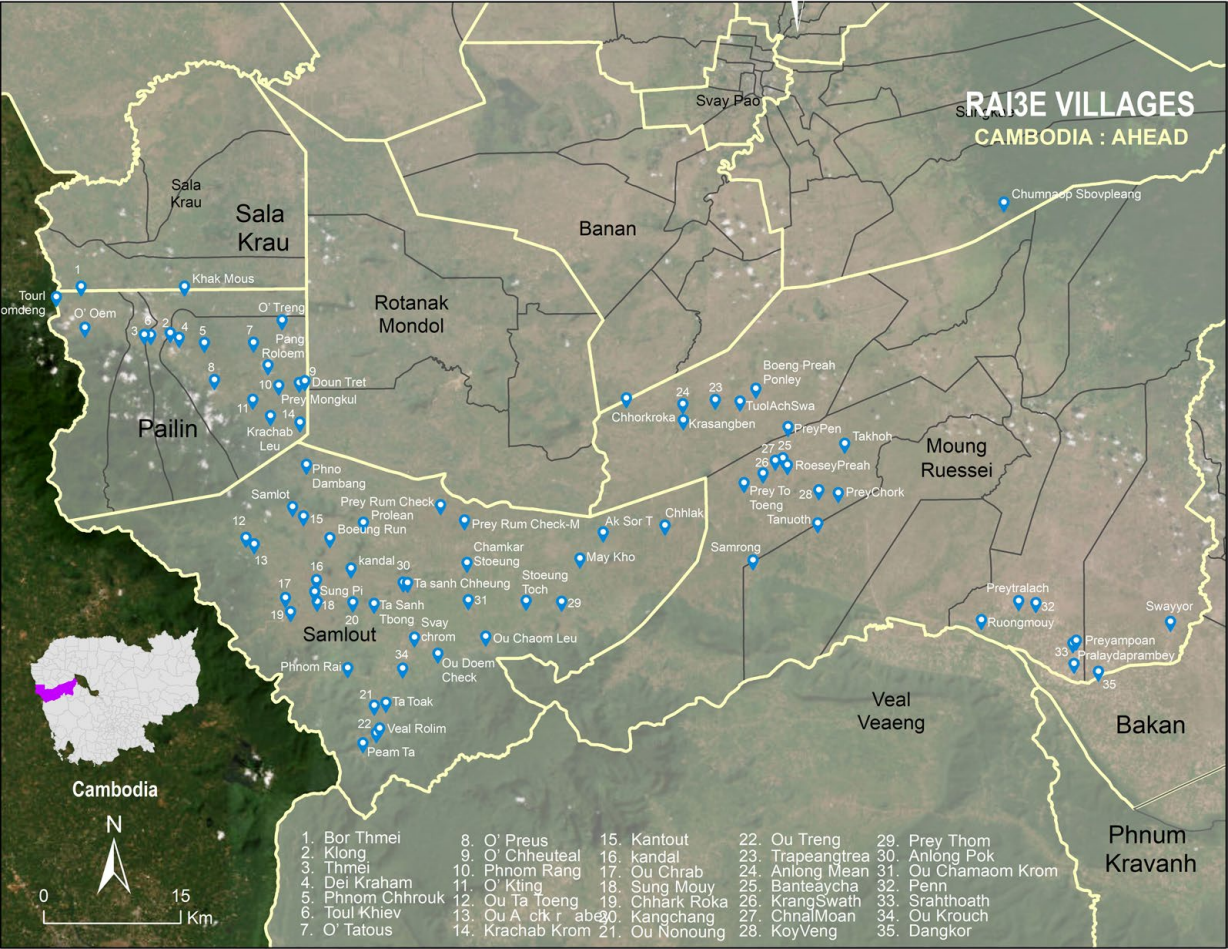
RAI3E study sites in Battambang province, western Cambodia

In Cambodia, the RAI3E operational research project included two studies, Work Package A, a stakeholder analysis of the roles of CHWs in Southeast Asia; and Work Package B, the operational research implemented in villages within Battambong province. The preliminary findings of Work Package B were presented in the meeting followed by discussions with the meeting participants. This was supervised by CNM and implemented by the Provincial Health Departments (PHD) in Battambang and Pailin and Action for Health and Development (AHEAD; a community development non-governmental organization), with technical support from the Mahidol Oxford Tropical Medicine Research Unit (MORU). The work was conducted in 82 villages in Battambang and Pailin provinces in western Cambodia and evaluated a range of interventions that included the use of novel rapid diagnostic test kits for febrile illness and health education modules (Fig. 2). Over a period of 18 months, VMWs were trained and provided with background knowledge of the diseases and the instructions for the use of test kits and their interpretations.

**Fig. 3:**
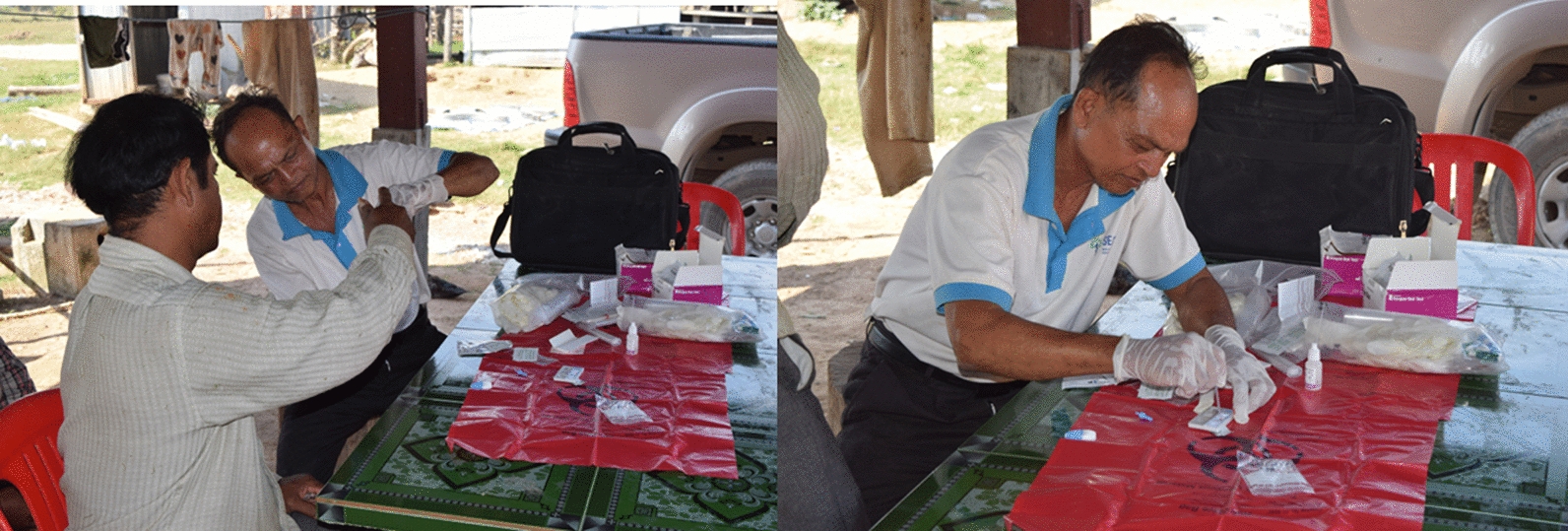
A VMW uses a Dengue Duo Rapid diagnostic test on a patient in the village

In August 2023, a meeting was held with the main objective to review the specific VMW roles and activities evaluated by the RAI-funded operational research in Cambodia and to discuss national and regional context, operational requirements, and conditions for implementation. For this, 20 participants convened at Battambang PHD representing stakeholder organizations that included the President’s Malaria Initiative (PMI), University Research Company (URC), United Nations Office for Project Services (UNOPS), Clinton Health Access Initiative (CHAI), the Cambodia Malaria Elimination Project (CMEP), and United States Agency for International Development (USAID). This report summarizes the discussions at the meeting. The meeting discussions were accompanied by the findings presented by the RAI3E investigators, and relevant partners who shared experience around potentials of expanding the roles of VMWs. In this report, the results of the meeting are presented based on the major topics that were extensively discussed, and were mostly focused on RAI3E findings, their practicalities and relevance to policy (Fig. 3).

## New diagnostics for village malaria workers

As a part of Work Package B, new diagnostics were offered to VMWs by AHEAD under the supervision of CNM and supported technically by MORU. A total of 105 VMWs were trained in 82 villages in the use of new diagnostics. These included dengue antigen–antibody RDTs (Additional file 1), combined malaria/CRP tests, and multiplexed biosensors (Additional file 2). Multiplexed biosensors were designed to test malaria (four *Plasmodium* species, using pLDH and HRP2), dengue virus, zika virus, chikungunya virus, leptospirosis, *Rickettsia typhi*, *Burkholderia pseudomallei*, and *Orientia tsutsugamushi*. [24]. The full report on the deployment of novel RDTs is under preparation (Visser et al. 2023). In brief, the preliminary results showed that 1511 malaria/CRP RDTs were performed by VMWs, of which 58 (3.8%) were positive (55 positive for raised CRP and three positives for malaria). A total of 917 dengue RDTs were performed by VMWs, of which 34 patients tested positive, 28 (3.1%) for NS1/IgM (representing an acute infection), and 6 (0.7%) were IgG positive only (representing past infections). All patients with positive results for raised CRP or acute dengue were referred to the local health centres for further management, and all malaria cases were managed by VMWs with or without onward referral based on existing practices. No deaths were recorded during the study among the enrolled population.

Work Package B aimed to explore the feasibility of point-of-care tests to diagnose acute febrile illnesses in primary care settings. As a part of the study, workshops and focus group discussions were held at nine health centres to explore the practicalities related to multiplexed biosensors, malaria/CRP, and dengue antigen–antibody tests [24]. In Battambang, some barriers to deploying the new diagnostics were reported. There were technical difficulties and concerns over the increased time and material requirements, and the need to draw large blood volumes. Some features were inconvenient, for instance, the number of steps and multiple sampling tools, buffers, and wells (leading to errors during test performance). RDTs could be improved if they were labelled better, for example, if they were equipped with distinct colours/numbers and in the local language. This was a limitation in deploying them to VMWs who lack the training of nurses and laboratory technicians. There was dissatisfaction from patients regarding the blood volume required for the dengue test (110 µl vs 15 µl for malaria/CRP), which was seen by some patients as potentially harmful and may affect the future acceptability of the test. Overall, dengue tests were not found to be convenient in comparison to malaria RDTs by the health centre staff [24]. In summary, the majority of VMWs could perform RDTs appropriately and follow management algorithms. VMWs were enthusiastic about alternative diagnostics despite their concerns and febrile patients continued to attend VMWs despite the low incidence of malaria, supporting ongoing elimination efforts. VMWs showed keen interest in new training, education, and additional roles. The RAI3E operational research received excellent support from local stakeholders and authorities.

## New health education packages for VMWs

A separate report on the health education modules has been published [25]. Briefly, VMWs were trained with four health education packages that included 1. Hygiene and sanitation; 2. Management of mild illnesses; 3. Disease surveillance and first aid; and 4. Immunization and antenatal care. The health education sessions were held at nine health centres. The practical recommendations from the evaluation of health education packages included simplification needed for health education materials to make them understandable to the communities, considering the low literacy level. VMWs recommended offering incentives to community members to compensate for their time and expenses when they attend health education sessions. Among the four packages, disease surveillance and first aid were preferred for utility and potential benefits to the community. Some minor challenges to delivering health education were identified including the perceived inadequacy of the incentives by community members when they participated in health education sessions. VMWs also perceived the inadequacy of the incentives when undertaking responsibilities particularly when they have other occupation essential to support their livelihoods. Some of the incentives suggested by community members included snacks or tangible items such as money, and/or soap. While some community members showed interest in attending such sessions, others had conflicting household responsibilities. Since males often had to prioritize the overall responsibilities related to livelihood, health education sessions were dominated by female participation.

## National and regional context of community health workers and malaria elimination

The preliminary findings of Work Package B were discussed to explore their alignment with the CNM’s policy on expanding VMWs’ roles and responsibilities. Meeting participants considered VMWs as the essential pillars of malaria elimination efforts in Cambodia and around the region [7]. CHWs have remained an essential pillar for early diagnosis and treatment of malaria, surveillance, and preventive activities. While CHWs are valuable resources for disease management in the community, they are also one of the most overlooked elements of health services, partly because they are not included in the formal health system structure or are simply peripheral. VMWs in Cambodia are a vital community resource for malaria case detection and management, and other malaria-related work. Nonetheless, with recent declines in total cases and proportional increase in vivax malaria, their role in vivax malaria management has been undergoing discussions. Following a presentation on expanding the roles of VMWs related to vivax malaria management that entailed point of care Glucose 6 Phosphate Dehydrogenase (G6PD) test using biosensor in a neighboring Kravanh district, participants shared some of the practical considerations such as the need to have more local and regional evidence on the application of biosensors including the improvement in product. The latter included discussions around the field feasibility of biosensors, such as their performance in hot and humid temperatures, and product related restrictions; and thus implied the need to have product competition to develop newer generation biosensors [26].

G6PD testing is recommended by the World Health Organization (WHO) before giving 8-aminoquinolones for radical cure of *P. vivax* but this has only been rolled out to health centres and hospitals in Cambodia. Administering 8-aminoquinolones in G6PD-deficient patients can cause haemolysis [27–30]. Although there are various options for G6PD enzyme assessment, point-of-care tests are seen as the most promising and are being implemented [31–36]. With these additional challenges, the roles and contributions of VMWs have been under discussion.

Maintaining VMWs in post is a major challenge. Additional resources for supervision and training are essential. Convening with VMWs monthly represents a major challenge for CNM, specifically because of the lack of incentives [37]. Meeting attendees accepted that VMWs are essential for malaria elimination and that it would be premature to phase out VMWs before malaria elimination is achieved.

## Discussion

The main objective of the meeting was to discuss the specific roles and responsibilities assigned to VMWs as a part of RAI-funded operational research in Cambodia and explore the feasibility and policy implications. The meeting was led by CNM and PHD in collaboration with AHEAD and MORU and was attended by 20 delegates from national and international non-governmental organizations in Battambong Provincial Department Hall. In addition, a few participants also attended the meeting online through Microsoft Teams. The meeting was structured by the agenda and presentations, followed by adequate time for discussions at the end of each presentation (Additional file 3). Below, the major discussions of the meetings, specifically on the practicalities of expanding roles of VMWs aligning with the current policy of CNM are reported.

In the current Cambodian context of declining malaria incidence, the discussion was mostly focused on the benefits of the RAI studies, and their policy implications. This RAI3E operational research has offered insights into the feasibility and practicalities of expanding the roles and responsibilities of VMWs. An operational study showed the potential of VMWs in executing additional roles, particularly in acquiring new knowledge and techniques about disease diagnosis and primary management. Although VMWs were able to use rapid diagnostic kits, the practical difficulty in using the dengue test was identified early on and may have important implications [24]. While any new techniques would inevitably require adequate and repeated training and supervision, some of the roles and responsibilities are difficult to implement and thus require careful consideration for the future [38]. In another operational study to explore the practicalities and feasibility of VMWs using biosensors to measure G6PD, the need for repeated training and supervision was found to be the key feature for optimal outcomes [5, 26, 39]. When these findings were discussed in the meeting, it was clear that health education-related roles and responsibilities were found to be more feasible for VMWs in future.

The sustainability of the VMW network can be key to reaching malaria elimination rapidly. An informative Southeast Asian case study comes from rural Myanmar where CHWs were associated with a sharp fall in malaria. The decrease in malaria was followed by a reduction in the number of febrile patients seeking care from CHWs. The subsequent expansion of their remit to general health care restored the uptake of patients attending CHWs and being tested for malaria [40]. In contrast, a large study with long-term surveillance elsewhere in Myanmar did not observe such a decline in attendance by febrile patients, despite a fall of ~ 90% in falciparum malaria cases over a period of 7 years [41, 42]. The continued attendance of malaria posts with dedicated malaria workers underscores the fact that sustaining malaria services at the community level is critical irrespective of the integration of their roles with additional diseases. In both scenarios with integrated community health workers or malaria-only workers, continued access to services, and community engagement were essential to strengthen early case detection and treatment when approaching malaria elimination [40–42].

The Cambodian malaria elimination framework outlines continued focus on case detection and treatment, accelerated efforts towards malaria elimination starting at the sub-national level, and transforming malaria surveillance and response as a core intervention [43, 44]. Despite the declining malaria trend, maintaining VMWs to continue their roles for malaria diagnosis and treatment even after the malaria has been eliminated has proven to be effective. For instance, in Sri Lanka, increased access to health services for malaria led to improved case detection and treatment during the pre-elimination phase. This was followed by sustained surveillance. Both measures were key to achieving malaria elimination [45, 46]. In Aneityum island of Vanuatu, community microscopists were sustained for case detection and treatment after malaria elimination and this approach was a key to the successful elimination of malaria [47, 48]. More recently, in El Salvador, the malaria CHW strategy consisted of (1) data-driven surveillance and response and (2) decentralization and expansion of community-based case management through a network of volunteer collaborators. The increased local capacity of community case management and surveillance was key to the achievement of malaria elimination [49]. Bhutan and Timor-Leste are progressing towards malaria elimination, and experience from these countries demonstrates that strengthening of community health services remains a core element [50].

**Fig. 4:**
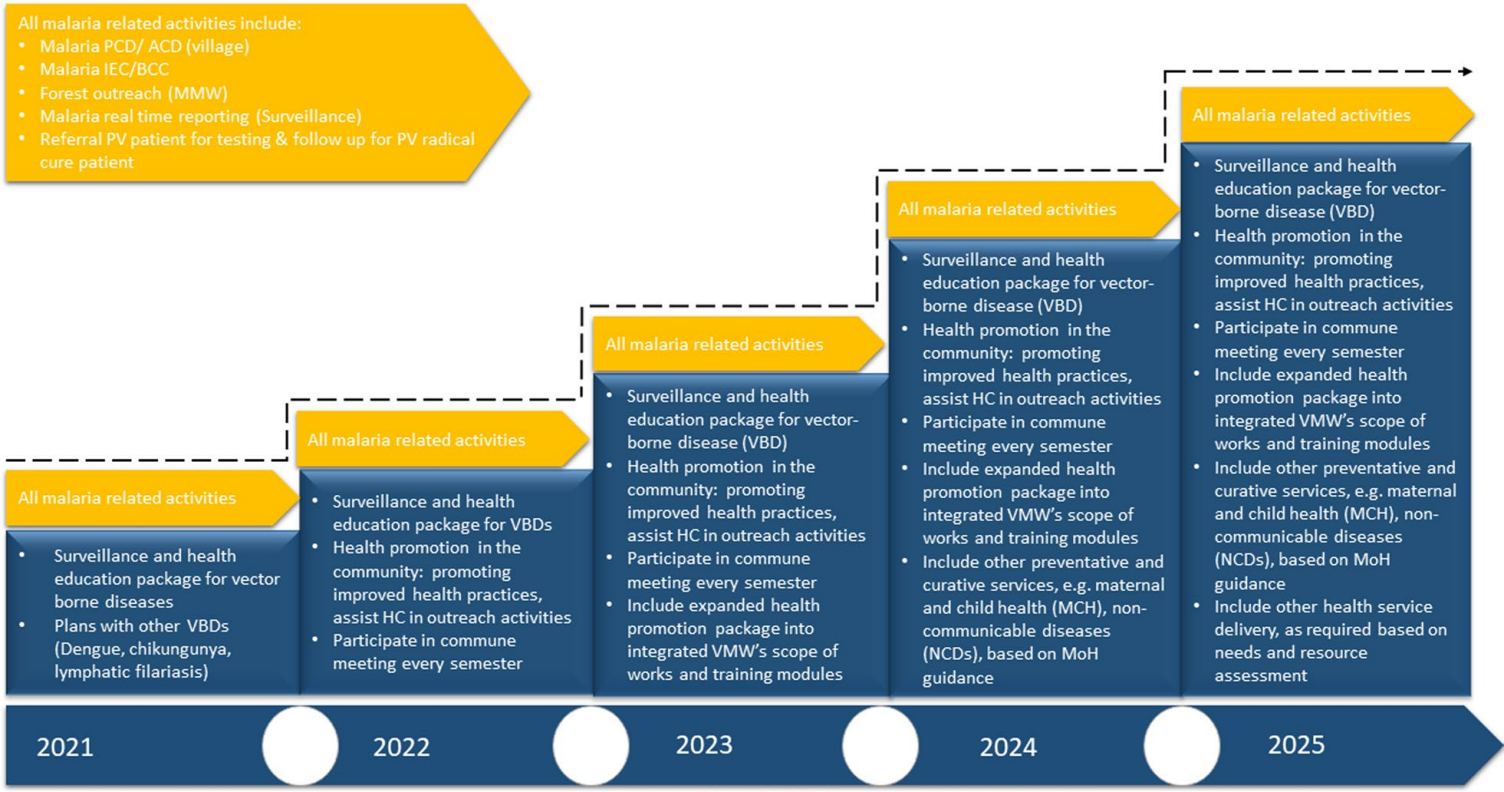
CNM roadmap for integrating VMW roles from 2021 to 2025. The figure is adapted from ‘*A roadmap to integration for village malaria workers 2021–225*’ by the National Centre for Parasitology, Entomology and Malaria Control 2021

Integrating VMWs into the health system through role expansion has been discussed in the policy framework by CNM and the integration plan has been underway since 2021. The integration was envisaged as a continuum until malaria elimination in 2025. The plan is to gradually integrate VMWs into community health care roles such as village health support groups to address other vector-borne diseases in the community. Nonetheless, their integration is an ongoing process and requires continuous adjustment. The roadmap for VMW role expansion and integration outlines a framework for how VMWs can sustain malaria-related activities in addition to taking on other responsibilities (Fig. 4).

Officially, VMWs are meant to be integrated into the community-based healthcare system, but not all VMWs are capable enough to take on additional roles. They are also affected by the community’s treatment-seeking dynamics and the endemicity of malaria in the village in which they work [13, 51]. The current national stratification, for example, categorizes villages based on the average reported annual parasite index (API), percentage of forest coverage, and distance from the village to health facilities. Based on the roadmap for VMWs integration of roles, those villages with higher risks of malaria and being located further away (> 5 km) from the health centre, along with having difficult road conditions, are recommended to maintain VMWs in their current roles in the province, while VMWs working in lower-risk villages may be proposed for role expansion [13]. This requires a careful evaluation of which VMWs are willing to, and are capable of, undertaking new roles and responsibilities, which is critical for integration. Part of the reason for the lack of definite conclusions on how to integrate the VMWs was also due to the ongoing development of the policy and the funding and resources it requires. It is difficult to imagine that VMWs could continue to deliver increasingly complex roles without adequate remuneration or better integration within the formal health system. A systematic review on how community health workers could take on integrated roles for community case management of malaria also identified challenges related to the need for sustainable financing, tailoring the training to address the identified gaps, and improving supply chain management [9, 52]. Integrated community case management (iCCM) of malaria, pneumonia and diarrhoea have been shown to be successful in the past and could possibly add optimism to Cambodia’s plan for adding roles related to vector-borne diseases (VBDs) [53, 54].

The integration of VMWs into the formal CHW system to support broader remits such as maternal and child health, non-communicable diseases, and even other infectious diseases requires engagement with various departments within the Ministry of Health. Subsequent policy formulation, resource management (e.g., funding), and importantly, training, supervision, and monitoring of the VMWs for additional responsibilities were considered complex health system undertakings. The WHO guideline on optimizing CHW programmes for integrated roles clearly outlines the need to embed CHW programmes in health systems and align them with broader national health and health workforce policies to optimize the quality of provided services [55]. Currently, among considerations for expanding the roles of VMWs are adding VBDs that include dengue, chikungunya, lymphatic filariasis, soil-transmitted helminths, and schistosomiasis. Integrating these VBDs alone will also require a blend of evidence, resources for training and supervision, and ultimately adaptation of policy based on the preliminary implementation [56].

Policy formulation is a complex and iterative process that often entails balancing the trade-offs between the available evidence at local, regional, and national levels, operational characteristics of launching a programme, budgetary feasibilities, interests and priorities for a policy against other diseases, and, ultimately, public relevance and acceptability [57, 58]. Future studies are critical to monitor policy implementation and evaluation. The current policy overseeing the integration of VMWs and Village Health Support Groups (VHSGs) requires more time, resources and adjustments in the future. In Myanmar, the integration of previous cadres of CHWs with new roles has been criticized mostly because the integration of roles (additional disease responsibilities) was carried out based on a vertical governance system alone rather than assessing operational feasibilities [59]. Integrating roles and responsibilities warrants utilization of all the stages laid out in policy cycles introducing the policy agenda for initial discussion, its design, and decision-making, followed by implementation, evaluation, and adaptation [60].

## Conclusions

Meeting participants agreed on most of the findings of the RAI Work Package B with the following considerations. VMWs were deemed highly motivated to undertake new training, receive education, and expand their roles. The majority VMWs could perform RDTs and take appropriate action but RDTs should be user-friendly, selected for locally circulating pathogens, and result in an actionable output within the context of the VMW-patient encounter. Disease-specific education and simple management algorithms must accompany the RDT rollout. Health education was considered as a promising and feasible new role for VMWs. VMWs could be integrated with other CHW programmes and if feasible this may be an important step in ensuring the sustainability of community-based malaria management through the VMW network. Additional pragmatic monitoring and evaluation would be beneficial if VMWs take on new roles.

### Supplementary Information

**Additional file 1. **STANDARD Q Dengue Duo Rapid Diagnostic Kits.

**Additional file 2. **STANDARD Malaria CRP Duo Rapid Diagnostic Kits.

**Additional file 3. **Organization of meeting discussions.

## Data Availability

The data is available upon request to the Mahidol Oxford Tropical Medicine Research Unit Data Access Committee (http://www.tropmedres.ac/data-sharing) complying with the data access policy (http://www.tropmedres.ac/_asset/file/data-sharing-policy-v1-0.pdf).
