# Supplementary material for: Expanding the roles of community health workers to sustain programmes during malaria elimination: a meeting report on operational research in Southeast Asia
Source: Malar J. 2024 Jan 2;23:2. doi: 10.1186/s12936-023-04828-4 (PMC10759643; doi:10.1186/s12936-023-04828-4)
Supplement: Supplementary file 1 — Additional file 1. STANDARD Q Dengue Duo Rapid Diagnostic Kits. [file 12936_2023_4828_MOESM1_ESM.pdf]

Quality approved by SD BIOSENSOR / For *in vitro* diagnostics use only

STANDARD Q

Dengue Duo

STANDARD™ Q Dengue Duo Test

PLEASE READ BACK PAGE CAREFULLY BEFORE YOU PERFORM THE TEST
VEUILLEZ LIRE ATTENTIVEMENT LE VERSO DE LA PAGE AVANT D'EFFECTUER LE TEST
LEA ATENTAMENTE LA PÁGINA POSTERIOR ANTES DE REALIZAR LA PRUEBA
LEIA CUIDADOSAMENTE O VERSO ANTES DE REALIZAR O TESTE

STANDARD™

Kit contents / Contenu du kit / Contenido del kit / Conteúdo dos kits

STANDARD Q Dengue Duo Test Kit contents the followings.

No.	Component
①	Test device / Dispositif de test / Dispositivo de prueba / Dispositivo de teste
②	Assay diluent (2ml) / Diluant d'essai (2ml) / Diluyente de ensayo (2ml) / Diluente de análise (2ml)
③	Sample collector [Disposable dropper (100μl)] / Collecteur d'échantillons [Compte-gouttes jetables (100μl)] / Colector de muestra [Gotero desechable (100 μl)] / Coletor de amostra [Conta-gotas descartável (100μl)]
④	Sample collector [STANDARD™ Ezi tube+(10μl)] / Collecteur d'échantillons [STANDARD™ Ezi tube + (10μl)] / Colector de muestra [STANDARD™ Ezi tube+(10μl)] / Coletor de amostra [STANDARD™ Ezi tubo+(10μl)]
⑤	Instructions for use / Mode d'emploi / Instrucciones de uso / Instruções de uso

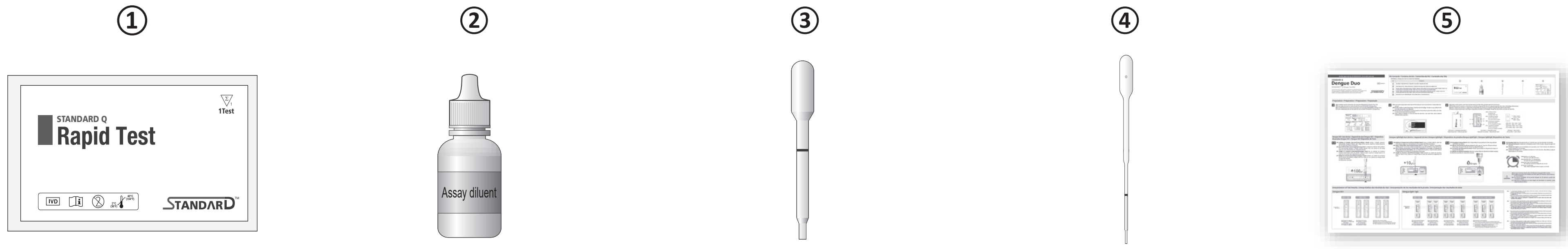

Preparation / Préparation / Preparación / Preparação

1 [EN] Carefully read the instruction for using the STANDARD Q Dengue Duo Test.
[FR] Lisez attentivement le mode d'emploi du test STANDARD Q Dengue Duo.
[ES] Lea atentamente las instrucciones para usar la prueba STANDARD Q Dengue Duo.
[PT] Leia cuidadosamente as instruções de uso do teste STANDARD Q Dengue Duo.

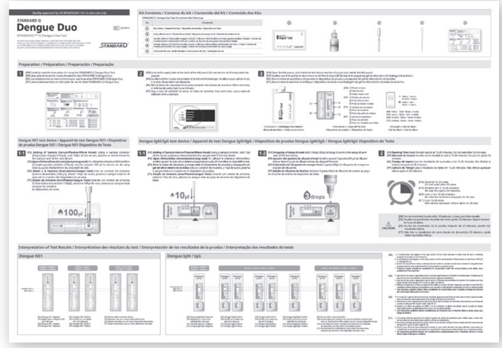

2 [EN] Look at the expiry date at the back of the foil pouch. Do not use the kit, if expiry date has passed.
[FR] Veuillez vérifier la date d'expiration à l'arrière de l'emballage. Veuillez ne pas utiliser le kit, si la date d'expiration est dépassée.
[ES] Mire la fecha de caducidad en la parte posterior de la bolsa de aluminio. Utilice otro lote, si la fecha de caducidad ha terminado.
[PT] Confira a data de validade no verso da bolsa de alumínio. Caso a data de validade tenha expirado, use outro lote.

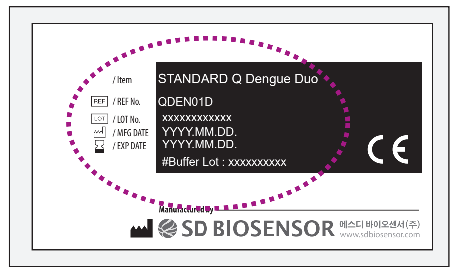

3 [EN] Open the foil pouch, and check the test device and the silica gel pack within the foil pouch.
[FR] Veuillez ouvrir le sachet en aluminium et vérifiez le dispositif de test et le paquet de gel de silice dans d'emballage d'aluminium.
[ES] Abra la bolsa de aluminio y compruebe el dispositivo de prueba y el paquete de gel de sílice dentro de la bolsa.
[PT] Abra a bolsa de alumínio e verifique o dispositivo de teste e a embalagem de sílica gel dentro da bolsa de alumínio.

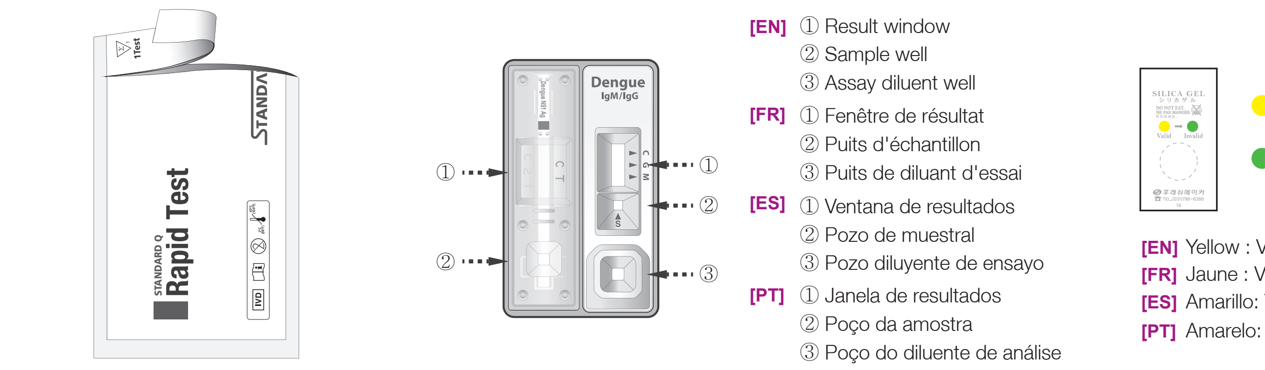

[EN] ① Result window
② Sample well
③ Assay diluent well

[FR] ① Fenêtre de résultat
② Puits d'échantillon
③ Puits de diluant d'essai

[ES] ① Ventana de resultados
② Pozo de muestral
③ Pozo diluyente de ensayo

[PT] ① Janela de resultados
② Poço da amostra
③ Poço do diluente de análise

Yellow / Jaune / Amarillo / Válido
Green / Vert / Verde / Inválido

[EN] Yellow : Valid / Green : Invalid
[FR] Jaune : Valide / Vert : Invalide
[ES] Amarillo: Válido / Verde: Inválido
[PT] Amarelo: Válido / Verde: Inválido

<Foil pouch> / <Emballage d'aluminium>
<Bolsa de aluminio> / <Bolsa de alumínio>

<Test device> / <Dispositif de test>
<Dispositivo de prueba> / <Dispositivo de teste>

<Silica gel> / <Gel de silice>
<Gel de silice> / <Sílica gel>

Test Procedure / Procédure de test / Procedimiento de la prueba / Procedimento teste

Dengue NS1 test device / Dispositif de test Dengue NS1 / Dispositivo de prueba Dengue NS1 / Dengue NS1 Dispositivo de Teste

1-1 [EN] **Adding of Sample (Serum/Plasma/Whole blood)** Using a sample collector [Disposable dropper(100μl)], add 100μl of the serum, plasma or whole blood to the sample well of the test device.
[FR] **Ajout d'échantillon (sérum/plasma/sang total)** En utilisant le collecteur d'échantillon [Compte-gouttes jetables (100μl)], veuillez ajouter 100 μl du sérum, plasma ou sang total au puits d'échantillon du dispositif de test.
[ES] **Añada a la muestra (Suero/plasma/sangre total)** Use un colector de muestra [Gotero desechable (100 μl)], añadir 100μl de suero, plasma o sangre total en el pozo de la muestra del dispositivo de prueba.
[PT] **Adição da Amostra (Soro/Plasma/Sangue Total)** Usando um coletor de amostra [Conta-Gotas descartável (100μl)], adicione 100μl do soro, plasma ou sangue total ao poço de amostra do dispositivo de teste.

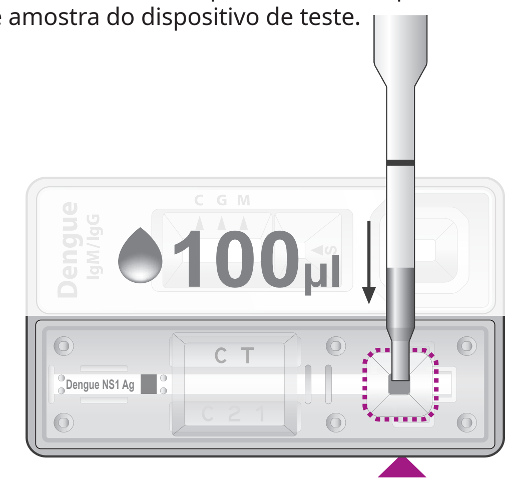

Dengue IgM/IgG test device / Dispositif de test Dengue IgM/IgG / Dispositivo de prueba Dengue IgM/IgG / Dengue IgM/IgG Dispositivo de Teste

1-1 [EN] **Adding of Sample (Serum/Plasma/Whole blood)** Using a sample collector, add 10μl of the serum, plasma or whole blood to the sample well of the test device.
[FR] **Ajout d'échantillon (sérum/plasma/sang total)** En utilisant le collecteur d'échantillon, veuillez ajouter 10 μl du sérum/plasma/sang total au puits d'échantillon du dispositif de test.
[ES] **Añada la bolsa de aluminio y compruebe el dispositivo de prueba y el paquete de gel de sílice dentro de la bolsa.** Use un Colector de muestra, y 10μl de suero, plasma o sangre total a la muestra en el dispositivo de prueba.
[PT] **Adição da Amostra (Soro/Plasma/Sangue Total)** Usando um coletor de amostra, adicione 10μl do soro, plasma ou sangue total ao poço de amostra do dispositivo de teste.

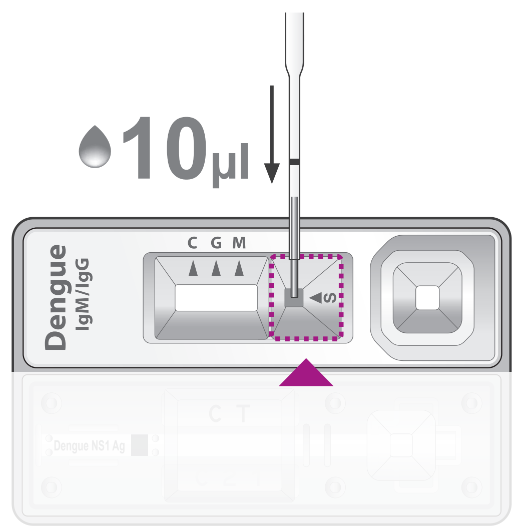

1-2 [EN] **Dropping of Assay diluent** Add 3 drops (90μl) of assay diluent to the assay diluent well of the test device.
[FR] **Ajouter des gouttes du diluant d'essai** Veuillez ajouter 3 gouttes (90 μl) de diluant d'essai dans le puits de diluant d'essai du dispositif de test.
[ES] **Colocación del diluyente de ensayo** Añada 3 gotas (90μl) de diluyente de ensayo en el pozo del diluyente.
[PT] **Adição do diluente de Análise** Adicione 3 gotas (90μl) do diluente de análise ao poço de diluente de análise do dispositivo de teste.

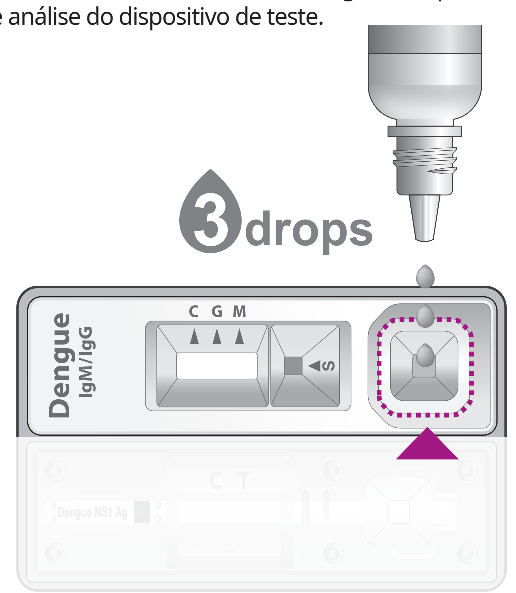

2 [EN] **Reading Time** Read the test results at 15-20 minutes. Do not read after 20 minutes.
[FR] **Temps de résultat** Veuillez lire le résultat du test à 15-20 minutes. Ne pas lire après 20 minutes.
[ES] **Tiempo de espera** Lea los resultados de la prueba a los 15-20 minutos. No efectúe la lectura después de 20 minutos.
[PT] **Tempo de Leitura** Leia o resultado do teste em 15-20 minutos. Não efetue a leitura após 20 minutos.

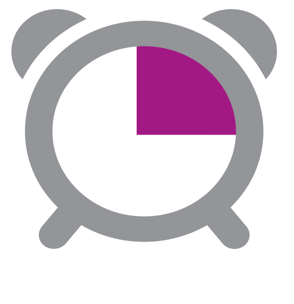

[EN] Read at 15-20 mins
Do not read after 20 mins
[FR] Veuillez lire à 15-20 minutes
Ne pas lire après 20 minutes
[ES] Leer a los 15-20 minutos
No efectúe la lectura después de 20 min
[PT] Leia em 15-20 minutos
Não efetue a leitura após 20 minutos

⚠ CAUTION

[EN] Do not read test results after 20 minutes, it may give false results.
[FR] Veuillez ne pas lire les résultats des tests après 20 minutes. Il peut donner de faux résultats.
[ES] No lea los resultados de la prueba después de 20 minutos, puede dar resultados falsos.
[PT] Não leia os resultados do teste depois de decorridos 20 minutos, os resultados podem ser falsos.

Interpretation of Test Results / Interprétation des résultats du test / Interpretación de los resultados de la prueba / Interpretação dos resultados do teste

Dengue NS1

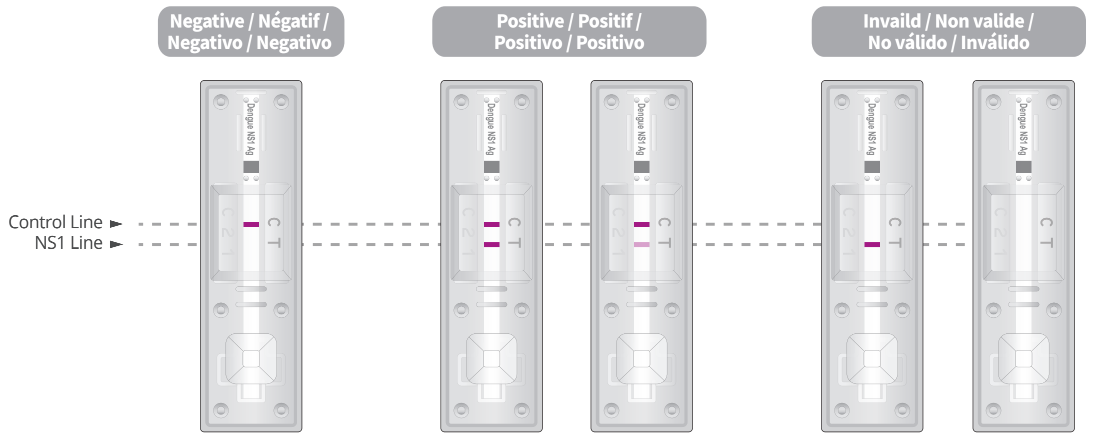

[EN] Dengue NS1 Negative
[FR] Négatif pour l'antigène NS1 de la dengue
[ES] Dengue NS1 negativo
[PT] Dengue NS1 Negativo

[EN] Dengue NS1 Positive
[FR] Positif pour l'antigène NS1 de la dengue
[ES] Dengue NS1 positivo
[PT] Dengue NS1 Positivo

[EN] Re-test with a new test device.
[FR] Retester avec un nouveau dispositif de test.
[ES] Repita la prueba con un nuevo dispositivo de prueba.
[PT] Teste novamente com um novo dispositivo de teste.

Dengue IgM / IgG

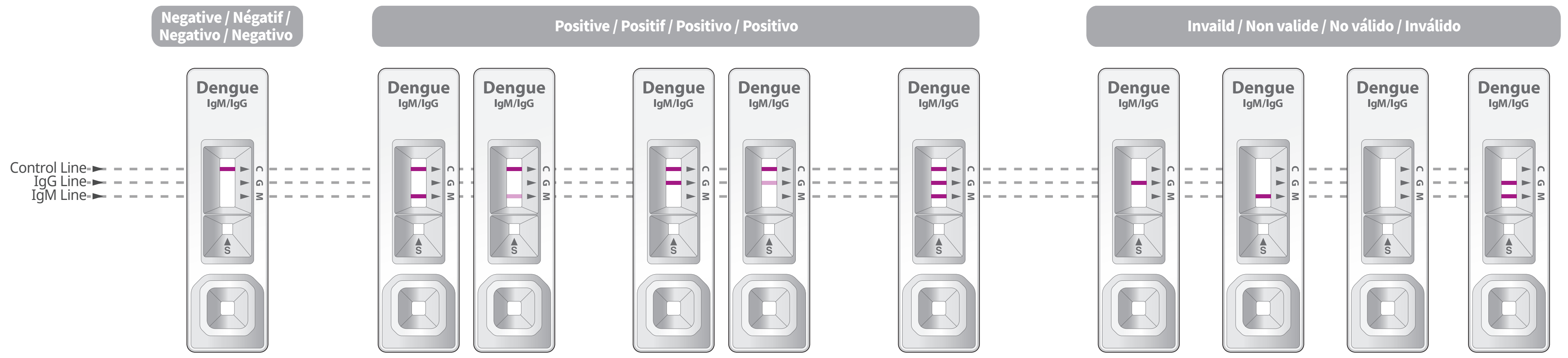

[EN] Dengue IgM/IgG Negative
[FR] Négatif pour l'anticorps IgM/IgG de la dengue
[ES] Dengue IgM/IgG negativo
[PT] Dengue IgM/IgG Negativo

[EN] Dengue IgM Positive
[FR] Positif pour l'anticorps IgM de la dengue
[ES] Dengue IgM positivo
[PT] Dengue IgM Positivo

[EN] Dengue IgG Positive
[FR] Positif pour l'anticorps IgG de la dengue
[ES] Dengue IgG positivo
[PT] Dengue IgG Positivo

[EN] Dengue IgM/igG Positive
[FR] Positif pour les anticorps IgM/IgG de la dengue
[ES] Dengue IgM/IgG positivo
[PT] Dengue IgM/IgG Positivo

[EN] Re-test with a new test device.
[FR] Aucune ligne de contrôle Il est recommandé de refaire le test avec un nouvel échantillon patient et un nouveau dispositif de test.
[ES] Se recomienda volver a realizar la prueba con una muestra del paciente y un dispositivo de prueba nuevos.
[PT] Recomenda-se a realização de novo teste com uma nova amostra do paciente e um dispositivo de teste novo.

[EN]
1. A colored band will appear in the top section of the result window to show that the test is working properly. This band is control line (C).
2. A colored band will appear in the lower section of the result window. These bands are test line of NS1 (T) or IgM/IgG (M, G).
3. Even if the control line is faint, or the test line isn't uniform, the test should be considered to be performed properly and the test result should be interpreted as a positive result.

* **Positive results should be considered in conjunction with the clinical history and other data available to the physician.**

[FR]
1. Une bande de couleur apparaîtra dans la section supérieure de la fenêtre de résultat pour montrer que le test fonctionne correctement. Cette bande est la ligne de contrôle (C).
2. Une bande de couleur apparaîtra dans la section inférieure de la fenêtre de résultat. Ces bandes sont des lignes de test de la dengue NS1 Ag (T) ou IgM / IgG (M, G).
3. Même si la ligne de contrôle qui apparaît est faible, ou la ligne de test n'apparaît pas plate, le test doit être considéré comme effectué correctement et le résultat du test doit être interprété comme un résultat positif.

* **Des résultats positifs doivent être considérés en conjonction avec l'histoire clinique et d'autres données disponibles pour le clinicien.**

[ES]
1. En la sección superior de la ventana de resultado aparecerá una línea de color para mostrar que la prueba está funcionando adecuadamente. Esta banda es la línea de control (C).
2. Una línea de color aparecerá en la sección inferior de la ventana de resultado. Estas bandas son líneas de prueba de dengue NS1 Ag (T) o IgM / IgG (M, G).
3. Incluso si la línea de prueba es débil o no es uniforme, se debe considerar que la prueba se realizó adecuadamente y se debe interpretar como un resultado positivo.

* **Los resultados positivos deben considerarse en conjunto con la historia clínica y otros datos que tenga el médico.**

[PT]
1. Aparecerá uma faixa colorida na seção superior da janela de resultado, indicando que o teste está funcionando corretamente. Essa faixa é a linha de controle (C).
2. Aparecerá uma faixa colorida na seção inferior da janela de resultado. Essa faixa é a linha de teste de dengue NS1 Ag(T) ou IgM/IgG (M,G).
3. Mesmo que a linha de teste esteja pouco visível, ou que a linha de teste não seja uniforme, o teste deve ser considerado corretamente executado e o resultado do teste deve ser interpretado como resultado positivo.

* **Os resultados positivos devem ser considerados conjuntamente com o histórico clínico e outros dados disponíveis para o médico.**

SD BIOSENSOR

English

Summary and Explanation

[Introduction]
Dengue viruses, transmitted by *Aedes aegypti* and *Aedes albopictus* mosquitoes, are widely distributed throughout the tropical and subtropical areas of the world. There are four known distinct serotypes of dengue virus (DEN-1, DEN-2, DEN-3 and DEN-4). Rapid and reliable tests for primary and secondary infections of Dengue are essential for patient management. An infected person experiences the acute symptoms of Dengue when there is a high level of the virus in the bloodstream. As the immune response fights the Dengue infection, the person's B cells begin producing IgMs and IgGs antibodies that are released in the blood and lymph fluid, where they recognize and neutralize the Dengue virus and viral molecules such as the Dengue non-structural protein 1 (NS1) antigen.

[Intended use]
STANDARD Q Dengue Duo is an immunochromatographic assay for the detection of dengue NS1 antigens and IgM/IgG antibodies to dengue in human serum, plasma, or whole blood samples. This test kit is for use in *in-vitro* diagnostic procedure for professional use.

[Test principle]
STANDARD Q Dengue Duo Test Kit has two devices. For NS1 Ag device, the rapid test membrane is coated with an anti-Dengue NS1 on the test line. The sample is added directly to the sample well and interacts with monoclonal anti-Dengue NS1-gold on the conjugate pad. This sample interacted with monoclonal anti-Dengue NS1-gold moves along membrane to the test line via capillary action to react with the anti-Dengue NS1. If NS1 antigen is present, a red line will appear at the test line. For IgM/IgG Ab device, monoclonal anti-human IgM and monoclonal anti-human IgG are immobilized at two individual test lines respectively (IgM/IgG line) on the membrane. Inactivated Dengue virus and monoclonal anti-Dengue-gold complex of pad release by adding assay diluent and react with anti-Dengue IgM or IgG. Human IgM and IgG in patient serum migrate and react with monoclonal anti-human IgM and IgG respectively on the membrane. If human anti-Dengue IgM or IgG exist in patient serum, the individual test line induced red colored band respectively, which means a positive test results. The red line at the control region should always appear if the assay is performed correctly.

[Kit contents]
① Test device ② Assay diluent (2ml)
③ Sample collector [STANDARD™ Ezi tube+(10µl)]
④ Sample collector [Disposable dropper (100µl)]
⑤ Instructions for use

Kit Storage and Stability

Store the kit at room temperature, 2-40°C / 36-104°F, out of direct sunlight. Kit materials are stable until the expiration date printed on the outer box. Do not freeze the kit.

Warnings

1. Do not reuse the test kit.
2. Do not use the test kit if the pouch is damaged or the seal is broken.
3. Do not use assay diluent of another lot.
4. Do not smoke, drink or eat while handling specimen.
5. Wear personal protective equipment, such as gloves and lab coats when handling kit reagents. Wash hands thoroughly when afterwards.
6. Clean up spills thoroughly using an appropriate disinfectant.
7. Handle all specimens as if they contain infectious agents.
8. Observe established recautions against microbiological hazards throughout testing procedures.
9. Dispose of all specimens and materials used to perform the test as bio-hazard waste. Laboratory chemical and bio-hazard wastes must be handled and discarded in accordance with all local, state, and national regulations.
10. Silica gel in foil pouch is to absorb moisture and keep humidity from affecting products. If the moisture indicating silica gel beads change from yellow to green, the test device in the pouch should be discarded.

Specimen collection and Preparation

[Serum]
1. If serum in the plain tube is stored in a refrigerator at 2-8°C/36-46°F, the specimen can be used for testing within 1 week after collection. Using the specimen in the long-term keeping more than 1 week can cause non-specific reaction. For prolonged storage, it should be at below -40°C/-40°F.
2. They should be brought to room temperature prior to use.

[Plasma]
1. If plasma in an anti-coagulant tube is stored in a refrigerator at 2-8°C/36-46°F, the specimen can be used for testing within 1 week after collection. Using the specimen in the long-term keeping more than 1 week can cause non-specific reaction. For prolonged storage, it should be at below -40°C/-40°F.
2. They should be brought to room temperature prior to use.

[Whole blood]
- **Capillary whole blood**
1. Capillary whole blood should be collected aseptically by fingertip.
2. Clean the area to be lanced with an alcohol swab.
3. Squeeze the end of the fingertip and pierce with a sterile lancet.
4. Collect the capillary whole blood to the black line of the capillary tube for the testing.
5. The capillary whole blood must be tested immediately after collection.
- **Venous Whole blood**
1. If venous whole blood in an anti-coagulant tube is stored in a refrigerator at 2-8°C/36-46°F, the specimen can be used for testing within 1-2 days after collection.
2. Do not use hemolyzed blood samples.

CAUTION
- Anticoagulants such as heparin or EDTA do not affect the test result.
- As known relevant interference, hemolytic sample, rheumatoid factors-contained sample and lipaemic, icteric sample can lead to impair the test results.
- Use separate disposable materials for each sample in order to avoid cross-contamination which can cause erroneous results.

Test Procedure

[Preparation]
1. Carefully read the instruction for using the STANDARD Q Dengue Duo Test.
2. Look at the expiry date at the back of the foil pouch. Do not use the kit, if expiry date has passed.
3. Open the foil pouch, and check the test device and the silica gel pack within the foil pouch.

[Test Procedure]
- **Dengue IgM/IgG test device**
1. Using a sample collector, add 10µl of the serum, plasma or whole blood to the sample well of the test device.
2. Add 3 drops (90µl) of assay diluent into the assay diluent well of the test device.
3. Read the test results at 15-20 minutes. Do not read after 20 minutes.
- **Dengue NS1 test device**
1. Using a sample collector [Disposable dropper(100µl)], add 100µl of the serum, plasma or whole blood to the sample well of the test device.
2. Read the test result at 15-20 minutes. Do not read after 20 minutes.

Limitation of the Test

1. The test procedure, precautions and interpretation of results for this test must be followed strictly when testing.
2. This test detects the presence of Dengue NS1 and Dengue IgMs/IgGs in the specimen and should not be used as the sole criteria for the diagnosis of Dengue virus infection.
3. Test results must be considered with other clinical data available to the physician.
4. For more accuracy of immune status, additional follow-up testing using other laboratory methods is recommended.
5. Neither the quantitative value nor the rate of Dengue NS1 Ag or anti-Dengue IgM/IgG concentration can be determined by this qualitative test.
6. Failure to follow the test procedure and interpretation of test results may adversely affect test performance and/or produce invalid results.

Performance Characteristics

- **The sensitivity and specificity of STANDARD Q Dengue Duo Test:** Total 860 samples were evaluated for sensitivity and specificity. The STANDARD Q Dengue Duo test kit got a high correlation with reference test (ELISA and RT-PCR).

Reference		STANDARD Q Dengue Duo (NS1)		Total Result
		Positive	Negative	
RT-PCR	Positive	184	14	198
	Negative	3	222	225
Total Result		187	236	423
Sensitivity: 184/198 (92.9%)<br>Specificity: 222/225 (98.7%)				

Reference		STANDARD Q Dengue Duo (IgM)		Total Result
		Positive	Negative	
ELISA	Positive	77	2	79
	Negative	12	346	358
Total Result		89	348	437
Sensitivity: 77/79 (97.5%)<br>Specificity: 346/358 (96.6%)				

Reference		STANDARD Q Dengue Duo (IgG)		Total Result
		Positive	Negative	
ELISA	Positive	140	4	144
	Negative	11	282	293
Total Result		151	289	437
Sensitivity: 140/144 (97.2%)<br>Specificity: 282/293 (96.2%)				

Bibliography

1. Dengue guidelines for diagnosis, treatment, prevention and control, World Health Organization, New Edition 2009.
2. Kliks SC, Nimmanitya S, Nisalak A, Burke DS, Evidence that maternal dengue antibodies are important in the development of dengue hemorrhagic fever in infants, Am J Trop Med Hyg Jan, 38(2):411-419, 1988.
3. Dengue haemorrhagic fever: Diagnosis, treatment, prevention and control, World Health Organization 2nd Edition, 1997.
4. Ludolfs D. et al. , Serological differentiation of infections with dengue virus serotypes 1 to 4 by using recombinant antigens, J Clin Microbiol, 40(11):4317-4320, 2002.
5. Matthew T. R. et al. Dengue virus pirates human platelets, Blood, 126(3):286-287, 2015.
6. Guzman M. G. et al. Dengue: A continuing global threat, Nat Rev Microbiol, 8:S7–S16, 2010.

Français

Résumé et explication

[Introduction]
Les virus de la dengue, transmis par les moustiques Aedes aegypti et Aedes albopictus, sont géographiquement très étendus dans les régions tropicales et subtropicales de la planète. Il existe quatre sérotypes connus différents du virus de la dengue (DEN-1, DEN-2, DEN-3 et DEN-4). Des tests rapides et fiables pour la détection des infections primaire et secondaire sont indispensables pour la prise en charge des patients. Une personne infectée présente les symptômes aigus de la dengue lorsqu'elle présente une grande concentration de virus dans le sang. Lorsque la réponse immunitaire lutte contre l'infection de dengue, les lymphocytes B de la personne commencent à produire des anticorps IgM et IgG qui sont libérés ans le sang et le liquide lymphatique, où ils reconnaissent et neutralisent les virus de la dengue et les molécules virales comme l'antigène NS1 de la dengue.

[Utilisation conforme]
Le test STANDARD Q Dengue Duo est un dosage immunochromatographique pour la détection des antigènes NS1 et anticorps IgM/IgG de la Dengue dans les échantillons de sérum, de plasma ou de sang total humain. Ce kit de test est réservé à une utilisation pour le diagnostic *in vitro*.

[Principe du test]
Le kit de test STANDARD Q Dengue Duo comprend deux dispositifs. Dans le dispositif pour la détection des antigènes NS1, la membrane est revêtue d'un anticorps spécifique du NS1 de la dengue dans la ligne de test et d'une IgY monoclonale anti-poulet dans la ligne de contrôle. Pour le dispositif dengue IgM / IgG Ab, qui ont la ligne «G», la ligne «M» et la «ligne de contrôle» pour la détection des anticorps IgM / IgG de la dengue. une IgM monoclonale anti-humaine et une IgG monoclonale anti-humaine sont immobilisées respectivement sur deux lignées de test (IgM, "M" / IgG, "G") et une IgG de chèvre anti-souris est immobilisée sur la ligne de contrôle de la nitrocellulose membrane. Les IgM et IgG humaines présentes dans le sérum du patient migrent et réagissent avec les IgM et IgG monoclonaux anti-humains respectivement sur la membrane. Le virus de la dengue inactivé et le complexe anticorps monoclonal anti-dengue-or sont libérés par l'ajout du diluant du test et réagissent avec les IgG ou les IgG anti-dengue. Si des IgG humaines ou des IgG humaines anti-dengue sont présentes dans le sérum du patient, une bande rouge apparaît dans la ligne de test respective et le résultat du test est positif. La bande de couleur rouge dans la zone de contrôle doit toujours apparaître si le test est correctement effectué.

[Kit contents]
① Dispositif de test ② Diluant d'essai (2ml)
③ Collecteur d'échantillons [Compte-gouttes jetables (100µl)]
④ Collecteur d'échantillons [tube STANDARD™ Ezi +(10µl)]
⑤ Mode d'emploi

Kit Storage and Stability

Conserver le kit à température ambiante, entre 2 et 40 °C / 36 et 104 °F, à l'abri de la lumière directe du soleil. Les matériaux du kit sont stables jusqu'à la date de péremption imprimée sur la boîte d'emballage. NE PAS CONGELER.

Avertissements

1. Veuillez ne pas réutiliser le kit de test.
2. Veuillez ne pas utiliser le kit de test si le l'emballage est endommagé ou si la bande de sécurité est cassée.
3. Veuillez ne pas utiliser le diluant d'essai d'un autre lot.
4. Veuillez ne pas fumer, boire ou manger pendant la manutention de l'échantillon.
5. Veuillez porter des équipements de protection individuelle tels que des gants et des blouses de laboratoire lors de la manipulation des réactifs du kit. Veuillez se laver les mains soigneusement après avoir fini les tests.
6. Veuillez nettoyer les déversements avec un désinfectant approprié.
7. Veuillez manipuler tous les échantillons comme s'ils contiennent des agents infectieux.
8. Veuillez observer les précautions établies contre les risques microbiologiques tout au long des procédures d'essai.
9. Veuillez jeter tous les échantillons et tous les matériaux utilisés durant le test en tant que déchets avec un risque biologique. Les déchets chimiques de laboratoire avec un risque biologique doivent être manipulés et mis au rebut conformément à toutes les réglementations locales, états et nationales.
10. Le gel de silice dans l'emballage en aluminium est destiné à absorber l'humidité et à empêcher l'humidité d'affecter les produits. Si l'humidité indiquent que les perles de gel de silice passent du jaune au vert, l'appareil d'essai dans l'emballage doit être jeté.

Collecter et preparer les echantillons

[Sérum]
1. Veuillez recueillir le sang veineux total dans le tube ordinaire disponible dans le commerce, Ne contenant PAS d'anticoagulants tels que l'héparine, l'EDTA ou le citrate de sodium, par ponction veineuse et laissez-le reposer pendant 30 minutes pour la coagulation sanguine puis centrifugez le sang pour obtenir l'échantillon de sérum du surnageant.
2. Si le sérum du tube ordinaire est conservé dans un réfrigérateur entre 2 et 8°C/36-46°F, l'échantillon peut être utilisé pour le test dans la première semaine suivante à la collecte. L'utilisation de l'échantillon à long terme pendant plus d'une semaine peut provoquer une réaction non spécifique. Pour un stockage prolongé, il doit être inférieur à -40°C/-40°F.
3. Ils doivent être portés à la température ambiante avant utilisation.

[Plasma]
1. Veuillez recueillir le sang veineux total dans le tube anticoagulant disponible dans le commerce comme l'héparine, l'EDTA ou le citrate de sodium par ponction veineuse et centrifuger le sang pour obtenir le spécimen plasmatique.
2. Si le plasma dans un tube anticoagulant est conservé dans un réfrigérateur entre 2 et 8°C/36-46°F, l'échantillon peut être utilisé pour le test dans la première semaine suivante à la collecte. L'utilisation de l'échantillon à long terme pendant plus d'une semaine peut provoquer une réaction non spécifique. Pour un stockage prolongé, il doit être inférieur à -40°C/-40°F.
3. Ils doivent être portés à la température ambiante avant utilisation.

[Sang total]
- **Sang total capillaire**
1. Le sang total capillaire doit être recueilli aseptiquement par le bout du doigt.
2. Veuillez nettoyer la zone avec un tampon alcoolisé.
3. Veuillez presser l'extrémité du bout du doigt et percer avec une lancette stérile.
4. Veuillez recueillir le sang total capillaire à la ligne noire du collecteur d'échantillon pour le test.
5. Veuillez tester le sang total capillaire immédiatement après la collecte.
- **Sang veineux total**
1. Veuillez recueillir le sang veineux total dans le tube anticoagulant disponible dans le commerce tel que l'héparine, l'EDTA ou le citrate de sodium par ponction veineuse.
2. Si le sang veineux total dans un tube anticoagulant est conservé dans un réfrigérateur à 2-8°C/36-46°F, l'échantillon peut être utilisé pour les tests dans les 1-2 jours après la collecte.
3. Veuillez ne pas utiliser d'échantillons de sang hémolysés.

Avertissement
- Les anticoagulants comme l'héparine ou l'EDTA n'ont aucun effet sur les résultats du test.
- Lorsque les échantillons sont hémolysés, lipémiques ou ictériques, ou contiennent des facteurs rhumatoïdes, les résultats du test risquent d'être altérés.
- Utiliser du matériel jetable différent pour chaque échantillon afin d'éviter toute contamination croisée susceptible de produire des résultats erronés.

Procédure de test

[Préparation]
1. Veuillez Lire attentivement le mode d'emploi du test STANDARD Q Dengue Duo.
2. Veuillez vérifier la date d'expiration à l'arrière de l'emballage. Veuillez ne pas utiliser le kit, si la date d'expiration est dépassée.
3. Veuillez ouvrir l'emballage en aluminium et vérifiez l'appareil de test et le pack de gel de silice dans l'emballage en aluminium.

[Procédure de test]
- **Appareil de test Dengue IgM/IgG**
1. En utilisant le collecteur d'échantillons, veuillez recueillir les 10 µl de sérum/plasma/sang total au puits d'échantillon du dispositif de test.
2. Veuillez ajouter 3 gouttes (90 µl) de diluant de l'essai dans le puits diluant de l'essai du dispositif de test.
3. Veuillez lire le résultat du test à 15-20 minutes. Ne pas lire après 20 minutes.
- **Appareil de test Dengue NS1**
1. En utilisant le collecteur d'échantillon [Compte-gouttes jetables (100µl)], veuillez ajouter 100 µl du sérum ou du sang total au puits d'échantillon du dispositif de test.
2. Veuillez lire le résultat du test à 15-20 minutes. Ne pas lire après 20 minutes.

Limite du test

1. La procédure de test, les précautions et l'interprétation des résultats de ce test doivent être scrupuleusement respectées pendant le test.
2. Ce test permet de détecter la présence d'antigènes NS1 de la dengue et d'anticorps IgG et/ou d'IgM de la dengue dans le spécimen et ne doit pas être utilisé comme seul critère pour le diagnostic d'une infection par le virus de la dengue.
3. Les résultats du test doivent être associés à d'autres données cliniques disponibles pour le médecin.
4. Pour préciser davantage l'état immunitaire, il est recommandé de réaliser d'autres tests de suivi fondés sur d'autres méthodes de laboratoire.

Caractéristiques de performance

- **Sensibilité et spécificité du test STANDARD Q Dengue Ag+Ab Duo:** Au total, 860 échantillons ont été évalués afin d'établir la sensibilité et la spécificité du test. Le kit de test STANDARD Q Dengue Duo a présenté une corrélation élevée avec le test de référence (ELISA et RT-PCR).

Reference		STANDARD Q Dengue Duo (NS1)		Total
		Positifs	Négatifs	
RT-PCR	Positifs	184	14	198
	Négatifs	3	222	225
Total		187	236	423
Sensibilité : 184/198 (92.9%)<br>Spécificité : 222/225 (98.6%)				

Reference		STANDARD Q Dengue Duo (IgM)		Total
		Positifs	Négatifs	
ELISA	Positifs	77	2	79
	Négatifs	12	346	358
Total		89	348	437
Sensibilité : 77/79 (97.5%)<br>Spécificité : 346/358 (96.6%)				

Reference		STANDARD Q Dengue Duo (IgG)		Total
		Positifs	Négatifs	
ELISA	Positifs	140	4	144
	Négatifs	11	282	293
Total		151	289	437
Sensibilité : 140/144 (97.2%)<br>Spécificité : 282/293 (96.2%)				

Bibliographie

1. Dengue guidelines for diagnosis, treatment, prevention and control, World Health Organization, New Edition 2009.
2. Kliks SC, Nimmanitya S, Nisalak A, Burke DS, Evidence that maternal dengue antibodies are important in the development of dengue hemorrhagic fever in infants, Am J Trop Med Hyg Jan, 38(2):411-419, 1988.
3. Dengue haemorrhagic fever: Diagnosis, treatment, prevention and control, World Health Organization 2nd Edition, 1997.
4. Ludolfs D. et al. , Serological differentiation of infections with dengue virus serotypes 1 to 4 by using recombinant antigens, J Clin Microbiol, 40(11):4317-4320, 2002.
5. Matthew T. R. et al. Dengue virus pirates human platelets, Blood, 126(3):286-287, 2015.
6. Guzman M. G. et al. Dengue: A continuing global threat, Nat Rev Microbiol, 8:S7–S16, 2010.

Español

Resumen y explicación

[Introducción]
Los virus del dengue, transmitidos por los mosquitos Aedes aegypti y Aedes albopictus, están ampliamente distribuidos en las zonas tropicales y subtropicales del mundo. Hay cuatro serotipos distintos conocidos del virus del dengue (DEN-1, DEN-2, DEN-3 y DEN-4). Las pruebas de detección rápida fiables para las infecciones primaria y secundaria del dengue son esenciales para el tratamiento de los pacientes. Una persona infectada experimenta los síntomas agudos del dengue cuando hay un nivel alto del virus en el torrente sanguíneo. A medida que la respuesta inmune combate la infección por dengue, las células B de la persona comienzan a producir anticuerpos IgM e IgG que se liberan en la sangre y el líquido linfático, donde reconocen y neutralizan el virus del dengue y moléculas virales como el antígeno NS1 del dengue.

[Uso previsto]
La prueba STANDARD Q Dengue Duo es un inmunoensayo cromatográfico para detectar los antígenos NS1 del Dengue y los anticuerpos IgM/IgG contra el Dengue en muestras de suero, plasma o sangre completa humana. Este kit de análisis es para uso en un procedimiento diagnóstico *in vitro*.

[Principio de prueba]
El kit de prueba STANDARD Q Dengue Duo incluye dos dispositivos. En el dispositivo de detección de antígeno NS1, la membrana está recubierta con un anticuerpo específico contra dengue NS1 en la línea de prueba y una IgY anti-pollo monoclonal en la línea de control. Para el dispositivo de dengue IgM / IgG Ab, que tiene la línea "G", la línea "M" y la "línea de control" para la detección de anticuerpos IgM / IgG contra el dengue. una IgM monoclonal antihumana y una IgG monoclonal antihumana se inmovilizan respectivamente en dos líneas de prueba (IgM, "M" / IgG, "G") y una IgG antiratón de cabra se inmoviliza en la línea de control de la membrana de nitrocelulosa. Las IgM e IgG humanas presentes en el suero del paciente migran y reaccionan con la IgM e IgG monoclonal anti-humano respectivamente en la membrana. El virus del dengue inactivado y el complejo de anticuerpos monoclonales anti-dengue gold se liberan mediante la adición del diluyente de prueba y reaccionan con IgG o IgG anti-dengue. Si está presente IgG humano o IgG humana anti-dengue en el suero del paciente, aparece una banda roja en la línea de prueba respectiva y el resultado de la prueba es positivo. La banda de color rojo en el área de control siempre debe aparecer si la prueba se realiza correctamente.

[Contenido del Kit]
① Dispositivo de prueba ② Diluyente de ensayo (2ml)
③ Colector de muestra [Gotero desechable (100µl)]
④ Colector de muestra [STANDARD™ Ezi tube+ (10 µl)]
⑤ Instrucciones de uso

Almacenamiento y estabilidad del kit

Almacenar el kit a temperatura ambiente a 2-40 °C/36-104 °F, lejos de la luz solar directa. Los materiales del kit son estables hasta la fecha de caducidad impresa en la caja exterior. NO CONGELAR.

Advertencias

1. No volver a usar el kit de prueba.
2. No utilice el kit de prueba si la bolsa está dañada o el sello está roto.
3. No use diluyente de ensayo de otro lote.
4. No fume, beba o coma mientras manipula la muestra.
5. Póngase un equipo de protección personal, como guantes y batas de laboratorio cuando manipule los reactivos del kit. Lávese bien las manos al terminar.
6. Limpie bien los vertidos con un desinfectante apropiado.
7. Manipule todas las muestras como si tuvieran agentes infecciosos.
8. Observe las precauciones establecidas contra los peligros microbiológicos durante el procedimiento de la prueba.
9. Elimine todas las muestras y material usados para realizar la prueba como residuos biodegradables. Los residuos químicos y de riesgo biológico de laboratorio deben ser manipulados y desechados de acuerdo con todas las regulaciones locales, estatales y nacionales.
10. El indicador de humedad con gel de sílice dentro de la bolsa de aluminio es para absorber la humedad y evitar que la humedad afecte a los productos. Si el paquete de gel de sílice dentro de la bolsa de aluminio es verde, los cristales deben ser de color Amarillo. Si el sílice es verde, deseche el dispositivo de prueba.

Recolección y preparación de muestras

[Suero]
1. Recoja la sangre total en el tubo plano disponible comercialmente que NO contenga anticoagulante como heparina, EDTA o citrato de sodio por venipunción y dejar reposar durante 30 minutos para la coagulación de la sangre y luego centrifugar la sangre para obtener la muestra de suero de sobrenadante.
2. Si el suero en el tubo plano es almacenado en un refrigerador a 2-8°C / 36-46°F, la muestra se puede utilizar para las pruebas en el plazo de una semana después de la recogida. El uso de la muestra a largo plazo de mantenimiento de más de una semana puede causar una reacción no específica. Para un almacenamiento prolongado, debe estar por debajo de -40°C / -40°F .
3. Debe ponerse a temperatura ambiente antes de usar.

[Plasma]
1. Recoja la sangre total venosa en el tubo anticoagulante comercialmente disponible tal como heparina, EDTA o citrato de sodio por punción venosa y centrifugar la sangre para obtener la muestra de plasma.
2. Si el plasma es almacenado en un tubo anticoagulante en un refrigerador a 2-8°C/ 36-46°F, la muestra puede usarse para pruebas dentro de una semana después de la recogida. Usar la muestra en un período largo de tiempo puede causar una reacción no específica. Para un almacenamiento prolongado, debe estar por debajo de -40°C/ -40°F.
3. Debe ponerse a temperatura ambiente antes de usar.

[Sang total]
- **Sangre total capilar**
1. La sangre total capilar debe ser recogida asépticamente con la yema del dedo.
2. Limpie el área a tratar con un hisopo con alcohol.
3. Apriete el extremo de la yema del dedo y pinche con una lanceta estéril.
4. Disponga la sangre entera total capilar a la línea negra del colector de muestra para la prueba.
5. La sangre total capilar debe ser probada inmediatamente después de su recogida.
- **Sangre venosa**
1. Recoja la sangre total venosa en el tubo anticoagulante comercialmente disponible tal como heparina, EDTA o citrato de sodio por punción venosa.
2. Si la sangre total venosa es almacenada en un tubo anticoagulante en un refrigerador a 2-8°C/ 36-46°F, la muestra puede usarse para pruebas dentro de 1-2 días después de la recogida.
3. No utilice muestras de sangre hemolizadas.

PRECAUCIÓN
- Los anticoagulantes como heparina o EDTA no afectan al resultado de la prueba.
- Las muestras hemolíticas, o con factores reumatoides, o lipémicas e ictéricas pueden desvirtuar los resultados de la prueba, ya que se trata de importantes interferencias conocidas.
- Utilice materiales desechables separados para cada muestra a fin de evitar una contaminación cruzada que pueda provocar resultados erróneos.

Procedimiento de la prueba

[Preparación]
1. Lea detenidamente las instrucciones para usar la Prueba STANDARD Q Dengue Duo.
2. Mire la fecha de caducidad en la parte posterior de la bolsa de aluminio. No use el kit, si la fecha de caducidad ha pasado.
3. Abra la bolsa de aluminio y comprobar el dispositivo de la prueba y el paquete de gel de sílice dentro de la bolsa de aluminio.

[Procedimiento de la prueba]
- **Dispositivo de prueba Dengue IgM/IgG**
1. Use un colector de muestra, añadir 10µl de suero, plasma o sangre total al pozo de la muestra del dispositivo de prueba.
2. Añada 3 gotas (90µl) de diluyente de ensayo dentro del pozo de diluyente de ensayo del dispositivo de prueba.
3. Lea el resultado de la prueba a los 15-20 minutos. No efectúe la lectura después de 20 minutos.
- **Dispositivo de prueba Dengue NS1**
1. Use un colector de muestra [Gotero desechable (100µl)], añadir 100µl del suero, plasma o sangre total al pozo de muestra del dispositivo de prueba.
2. Lea los resultados de la prueba a los 15-20 minutos. No efectúe la lectura después de 20 minutos.

Limitación de la prueba

1. Al realizar pruebas, es necesario seguir estrictamente los procedimientos, precauciones e interpretación de resultados de la prueba.
2. Esta prueba detecta la presencia del NS1 del dengue y los IgG o IgM contra el dengue en la muestra; no debe utilizarse como único criterio al diagnosticar una infección por el virus del dengue.
3. Los resultados de la prueba deben valorarse junto con otros datos clínicos disponibles para el médico.
4. Para obtener una mayor precisión en cuanto al estado de inmunidad, se recomienda realizar pruebas de seguimiento adicionales utilizando otros métodos de laboratorio.

Características de rendimiento

- **Sensibilidad y especificidad de la prueba STANDARD Q Dengue Duo:** Se evaluaron la sensibilidad y la especificidad en un total 860 muestras. El kit de prueba STANDARD Q Dengue Duo obtuvo una notable correlación con la prueba de referencia (ELISA y PCR en tiempo real).

Reference		STANDARD Q Dengue Duo (NS1)		Total
		Positivo	Negativo	
RT-PCR	Positivo	184	14	198
	Negativo	3	222	225
Total		187	236	423
Sensibilidad : 184/198 (92.9%)<br>Especificidad : 222/225 (98.6%)				

Reference		STANDARD Q Dengue Duo (IgM)		Total
		Positivo	Negativo	
ELISA	Positivo	77	2	79
	Negativo	12	346	358
Total		89	348	437
Sensibilidad : 77/79 (97.5%)<br>Especificidad : 346/358 (96.6%)				

Reference		STANDARD Q Dengue Duo (IgG)		Total
		Positivo	Negativo	
ELISA	Positivo	140	4	144
	Negativo	11	282	293
Total		151	289	437
Sensibilidad : 140/144 (97.2%)<br>Especificidad : 282/293 (96.2%)				

Bibliografía

1. Dengue guidelines for diagnosis, treatment, prevention and control, World Health Organization, New Edition 2009.
2. Kliks SC, Nimmanitya S, Nisalak A, Burke DS, Evidence that maternal dengue antibodies are important in the development of dengue hemorrhagic fever in infants, Am J Trop Med Hyg Jan, 38(2):411-419, 1988.
3. Dengue haemorrhagic fever: Diagnosis, treatment, prevention and control, World Health Organization 2nd Edition, 1997.
4. Ludolfs D. et al. , Serological differentiation of infections with dengue virus serotypes 1 to 4 by using recombinant antigens, J Clin Microbiol, 40(11):4317-4320, 2002.
5. Matthew T. R. et al. Dengue virus pirates human platelets, Blood, 126(3):286-287, 2015.
6. Guzman M. G. et al. Dengue: A continuing global threat, Nat Rev Microbiol, 8:S7–S16, 2010.

Português

Sumário e explicação

[Introdução]
O vírus da dengue, transmitido pelo mosquito Aedes aegypti e Aedes albopictus, está amplamente difundido nas regiões áreas tropicais e subtropicais em nível global. Existem quatro sorotipos distintos do vírus da dengue conhecidos (DEN-1, DEN-2, DEN-3 e DEN-4). É essencial dispor de testes rápidos e confiáveis para detectar infecções primárias e secundárias pelo vírus da dengue para controle do paciente. Uma pessoa infectada apresenta sintomas agudos do vírus da dengue quando existe um elevado nível de vírus na corrente sanguínea. À medida que a resposta imunológica luta contra a infecção da dengue, as células B da pessoa começam a produzir anticorpos IgM e IgG, que são libertados no sangue e no fluido linfático, onde reconhecem e neutralizam o vírus da dengue e as moléculas virais como o antígeno NS1 do vírus da dengue.

[Uso pretendido]
O STANDARD Q Dengue Duo Test é um ensaio imunocromatográfico para detecção de antígenos NS1 e anticorpos IgM/IgG do vírus da dengue em amostras de sangue de soro, plasma ou sangue total humanos. Este kit de teste destina-se a ser usado em procedimentos de diagnóstico *in vitro*.

[Princípios do teste]
O kit de teste STANDARD Q Dengue Duo inclui dois dispositivos. No dispositivo de detecção de antígeno NS1, a membrana é revestida com um anticorpo específico NS1 de dengue na linha de teste e uma IgY monoclonal anti-galinha na linha de controle. Para o dispositivo IgM / IgG Ab dengue, que tem a linha "G", a linha "M" e a "linha de controle" para a detecção de anticorpos IgG / IgG de dengue. uma IgM monoclonal anti-humana e uma IgG monoclonal anti-humana são imobilizadas respectivamente em duas linhas de teste (IgM, "M" / IgG, "G") e uma IgG de cabra anti-camundongo é imobilizada na linha de controle da membrana de nitrocelulose. As IgM e IgG humanas presentes no soro do paciente migram e reagem com IgM e IgG monoclonais anti-humanos, respectivamente, na membrana. O vírus da dengue inativado e o complexo de anticorpo monoclonal de ouro anti-dengue são liberados pela adição do diluente de teste e reagem com IgG ou IgG anti-dengue. Se houver a presença de IgG humana ou IgG humana anti-dengue no soro do paciente, aparece uma faixa vermelha na linha de teste respectiva e o resultado do teste é positivo. A faixa de cor vermelha na área de controle aparecerá sempre que o teste for realizado corretamente.

[Conteúdo do kit]
① Dispositivo de teste ② Diluente de ensaio (2ml)
③ Coletor de amostra [Conta-gotas descartável (100µl)]
④ Coletor de amostra [STANDARD™ Ezi tubo+(10µl)]
⑤ Instruções de uso

Armazenamento e estabilidade do kit

Armazene o kit à temperatura ambiente, 2-40°C, protegido de luz solar direta. Os materiais do kit mantêm-se estáveis até ao fim do prazo de validade impresso na embalagem externa. NÃO CONGELAR.

Avisos

1. Não reuse o kit de teste.
2. Não use o kit de teste se a bolsa estiver danificada ou se o selo estiver violado.
3. Não use diluente de análise de outro lote.
4. Não fume, beba ou coma enquanto manuseia a amostra.
5. Use equipamento de proteção como luvas e aventais de laboratório para manusear os reagentes do kit.
6. Limpe com cuidado todos os respingos, usando desinfetantes apropriados.
7. Manuseie cuidadosamente todas as amostras, pois elas contêm agentes infecciosos.
8. Observe as precauções estabelecidas contra os perigos microbiológicos ao longo dos procedimentos de teste.
9. Descarte como resíduos com risco biológico todas e quaisquer amostras e materiais usados para realizar o teste. Produtos químicos de laboratório e resíduos com risco biológicos devem ser manuseados e descartados de acordo com todos os regulamentos locais, estatais e nacionais.
10. Um indicador de humidade com sílica gel dentro da bolsa de alumínio serve para absorver a humidade e evitar que esta afete os produtos. Se a embalagem de sílica gel dentro da bolsa de alumínio for verde, os cristais deverão ser amarelos. Se a sílica gel estiver verde, descarte o dispositivo de teste.

Coleta e preparação da amostra

[Soro]
1. Colete o sangue total por venipunção em um tubo simples comercial disponível que NÃO contenha anticoagulantes, como heparina, EDTA ou citrato de sódio e deixe repousar por 30 minutos para a coagulação do sangue. Em seguida, centrifugue o sangue para obter amostras de soro do sobrenadante
2. Se o soro no tubo simples for armazenado em um refrigerador a 2-8°C, a amostra pode ser usada para teste em até uma semana após a coleta. O uso da amostra em um prazo mais longo, por mais de uma semana, poderá causar uma reação não específica. No caso de um armazenamento prolongado, a amostra deverá permanecer a uma temperatura abaixo de -40°C / -40°F..
3. Antes de ser usada, a amostra deverá estar na temperatura ambiente.

[Plasma]
1. Colete o sangue total por venipunção no tubo anticoagulante comercial disponível como heparina, EDTA ou citrato de sódio e centrifugue o sangue para obter a amostra de plasma.
2. Se o soro no tubo anticoagulante for armazenado em um refrigerador a 2-8°C, a amostra pode ser usada para teste em até uma semana após a coleta. O uso da amostra em um prazo mais longo, por mais de uma semana, poderá causar uma reação não específica. No caso de um armazenamento prolongado, a amostra deverá permanecer a uma temperatura abaixo de -40°C / -40°F.
3. Antes de ser usada, a amostra deverá estar na temperatura ambiente.

[Sangue total]
- **Sangue total capilar**
1. O sangue total capilar deve ser colhido assepticamente pela ponta do dedo.
2. Limpe a área de punção com uma compressa com álcool.
3. Aperte a ponta do dedo e perfure com uma agulha esterilizada.
4. Colete o sangue total capilar até a linha preta do coletor da amostra.
- **Sangue total venoso**
1. Colete o sangue total venoso, por venipunção, no tubo anticoagulante comercial disponível como heparina, EDTA ou citrato de sódio.
2. Se o sangue total venoso no tubo anticoagulante for armazenado no frigorífico a 2-8°C / 36-46°F, a amostra pode ser usada para teste até 1-2 dias após a coleta.
3. Não use amostras de sangue hemolisadas.

ATENÇÃO
- Anticoagulantes como heparina ou EDTA não afetam o resultado do teste.
- Interferências relevantes conhecidas presentes em amostras hemolíticas, amostras com fatores reumatoides e amostras lipêmicas podem prejudicar os resultados do teste.
- Use materiais descartáveis separados para cada amostra, para evitar contaminação, que poderia causar erro nos resultados.

Procedimento de teste

[Preparação]
1. Leia atentamente as instruções de uso do teste STANDARD Q Dengue Duo.
2. Preste atenção na data de validade no verso da bolsa de alumínio. Não use o kit caso a data de validade esteja vencida.
3. Abra a bolsa de alumínio e verifique o dispositivo de teste e a embalagem de sílica gel dentro da bolsa de alumínio.

[Procedimento de Teste]
- **Dispositivo de teste Dengue IgM/IgG**
1. Usando um coletor de amostra, adicione 10µl de soro, plasma ou sangue total ao poço da amostra do dispositivo de teste.
2. Adicione 3 gotas (90µl) de diluente de análise no poço de diluente de análise do dispositivo de teste.
3. Leia o resultado do teste em 15-20 minutos. Não efetue a leitura após 20 minutos.
- **Dispositivo de teste Dengue NS1**
1. Usando um coletor de amostra [Conta-Gotas Descartável (100µl)], adicione 100µl de soro, plasma ou sangue total ao poço da amostra do dispositivo de teste.
2. Leia o resultado do teste em 15-20 minutos. Não efetue qualquer leitura após os 20 minutos.

Limitações do teste

1. Na realização do teste, é preciso seguir rigorosamente o procedimento, as precauções e a interpretação dos resultados do teste.
2. Este teste detecta a presença do antígeno NS1 da dengue e dos anticorpos IgM/IgG do vírus da dengue na amostra e não deve ser usado como único critério para o diagnóstico da infeção pelo vírus da dengue.
3. Os resultados do teste devem ser considerados juntamente com outros dados clínicos disponíveis para o médico.
4. Para determinar um estado imunológico mais preciso, recomenda-se a execução de testes adicionais de acompanhamento, usando outros métodos laboratoriais.

Caraterísticas de desempenho

- **A sensibilidade e especificidade do Teste STANDARD Q Dengue Duo:** Foram avaliadas 860 amostras no total quanto à sensibilidade e especificidade. O Kit de Teste STANDARD Q Dengue Duo obteve uma elevada correlação com o teste de referência (ELISA e RT-PCR).

Reference		STANDARD Q Dengue Duo (NS1)		Total
		Positivo	Negativo	
RT-PCR	Positivo	184	14	198
	Negativo	3	222	225
Total		187	236	423
Sensibilidade : 184/198 (92.9%)<br>Especificidade : 222/225 (98.6%)				

Reference		STANDARD Q Dengue Duo (IgM)		Total
		Positivo	Negativo	
ELISA	Positivo	77	2	79
	Negativo	12	346	358
Total		89	348	437
Sensibilidade : 77/79 (97.5%)<br>Especificidade : 346/358 (96.6%)				

Reference		STANDARD Q Dengue Duo (IgG)		Total
		Positivo	Negativo	
ELISA	Positivo	140	4	144
	Negativo	11	282	293
Total		151	289	437
Sensibilidade : 140/144 (97.2%)<br>Especificidade : 282/293 (96.2%)				

Bibliografia

1. Dengue guidelines for diagnosis, treatment, prevention and control, World Health Organization, New Edition 2009.
2. Kliks SC, Nimmanitya S, Nisalak A, Burke DS, Evidence that maternal dengue antibodies are important in the development of dengue hemorrhagic fever in infants, Am J Trop Med Hyg Jan, 38(2):411-419, 1988.
3. Dengue haemorrhagic fever: Diagnosis, treatment, prevention and control, World Health Organization 2nd Edition, 1997.
4. Ludolfs D. et al. , Serological differentiation of infections with dengue virus serotypes 1 to 4 by using recombinant antigens, J Clin Microbiol, 40(11):4317-4320, 2002.
5. Matthew T. R. et al. Dengue virus pirates human platelets, Blood, 126(3):286-287, 2015.
6. Guzman M. G. et al. Dengue: A continuing global threat, Nat Rev Microbiol, 8:S7–S16, 2010.

REF QDEN01D

CE IVD

EC REP **Authorized Representative**
MT Promedt Consulting GmbH
Altenhofstrasse 80 66386 St. Ingbert Germany
Phone : +49 6894 581020, Fax : +49 6894 581021

L23DEN3MLR4
Issue date: 2019.11

REF - Reference number - Numéro de référence - Número de referencia - Número de referência
IVD - In vitro Diagnostic - Diagnostic in vitro - Diagnóstico in Vitro - Diagnóstico in Vitro
- Consult instructions for Use - Consulter le mode d'emploi - Consultar instrucciones de Uso - Consultar instruções de uso
- Contains Sufficient for <n> Tests - Contient <n> tests - Contiene suficiente para pruebas <n> - Contém o suficiente para <n> testes
- Caution - Avertissement - Precaución - Atenção
- Do not re-use - Usage unique - No volver a usar - Não reutilizar
- To indicate the temperature limitations in which the transport package has to be kept and handled. - Veuillez bien conserver et manipuler le test pour indiquer les limites de température dans laquelle le pack de transport. - Indicar las limitaciones de temperatura en las que debe mantenerse y manipularse el paquete de transporte. - Indica as limitações de temperatura relativas a como deve ser manuseado durante transporte.
- Use by - Utiliser par - Usar por - Usado por
LOT - Batch code - Code du lot - Código de lote - Código do lote
CE - Fulfill the requirements of Directive 98/79/EC on in vitro diagnostic medical devices [illegible]
- Manufacturer - Fabricant - Fabricante - Fabricante
- Date of manufacture - Date de fabrication - Fecha de fabricación - Data de fabricação
- Keep away from sunlight - Tenir ce produit éloigné de la lumière du soleil - Evitar luz solar directa - Não utilize se a embalagem estiver danificada
- Do not use if packaging is damaged - Ne pas utiliser si l'emballage est endommagé - No utilizar si el empaque está dañado - Não utilize se a embalagem estiver danificada

Manufactured by **SD Biosensor, Inc.**
Head office: C-4th&5th, 16, Deogyeong-daero 1556beon-gil, Yeongtong-gu, Suwon-si, Gyeonggi-do, 16690, REPUBLIC OF KOREA
Manufacturing site: 74, Osongsaengmyeong 4-ro, Osong-eup, Heungdeok-gu, Cheongju-si, Chungcheongbuk-do, 28161, REPUBLIC OF KOREA
Contact e-mail: sales-MKT@sdbiosensor.com
Website: www.sdbiosensor.com
