# Supplementary material for: Expanding the roles of community health workers to sustain programmes during malaria elimination: a meeting report on operational research in Southeast Asia
Source: Malar J. 2024 Jan 2;23:2. doi: 10.1186/s12936-023-04828-4 (PMC10759643; doi:10.1186/s12936-023-04828-4)
Supplement: Supplementary file 2 — Additional file 2. STANDARD Malaria CRP Duo Rapid Diagnostic Kits. [file 12936_2023_4828_MOESM2_ESM.pdf]

Quality approved by SD BIOSENSOR / For *in vitro* diagnostics use only

STANDARD Q

REF QMAL01D

Malaria/CRP Duo

STANDARD™ Q Malaria/CRP Duo Test

PLEASE READ BACK PAGE CAREFULLY BEFORE YOU PERFORM THE TEST

STANDARD™

Kit Contents

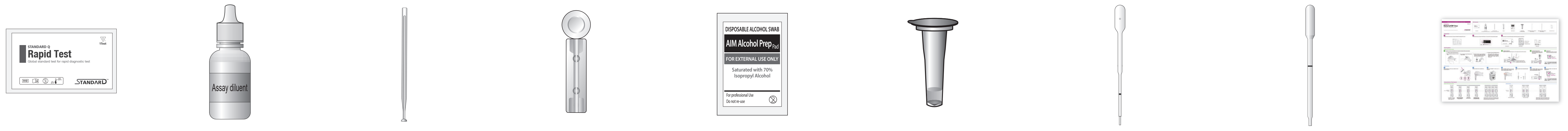

Test device | Assay diluent for Malaria P.f/Pan Ag Test device | Inverted cup (5µl) for Malaria P.f/Pan Ag Test device | STANDARD™ Lancet | STANDARD™ Alcohol swab | Assay diluent for CRP Test device | STANDARD™ Ezi tube+ (10µl) for CRP Test device | Disposable dropper (100µl) for CRP Test device | Instructions for use

Preparation

1

Carefully read the instructions for using the STANDARD Q Malaria/CRP Duo Test.

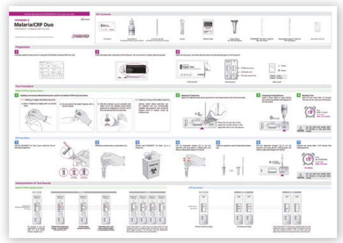

2

Check the expiry date at the back of the foil pouch. Do not use the kit, if expiry date has passed.

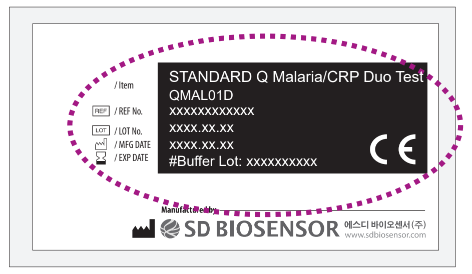

3

Open the foil pouch, and check the test device and the silica gel pack in the foil pouch.

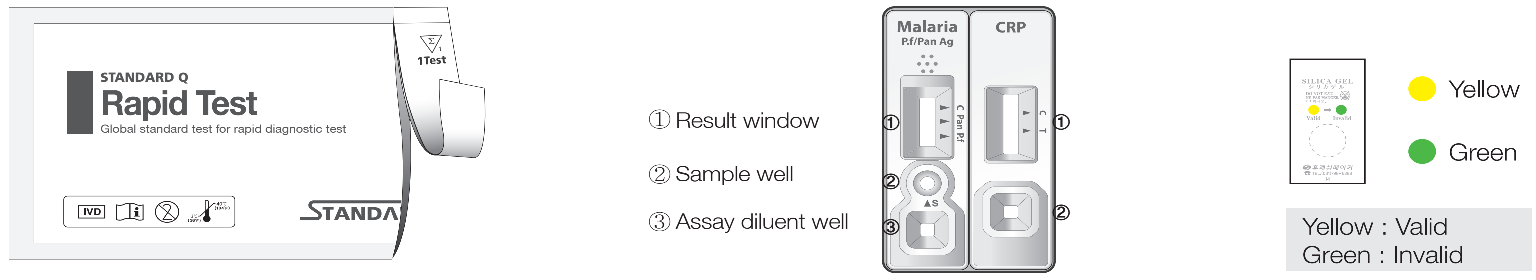

<Foil pouch> <Test device> <Silica gel>

Test Procedure

Malaria P.f/Pan Ag test device

1 **Capillary and venous whole blood can be used for the Malaria P.f/Pan Ag Test device**

1-1. Collecting of Capillary Whole Blood Specimen

① Clean a fingertip by wiping with an alcohol swab.

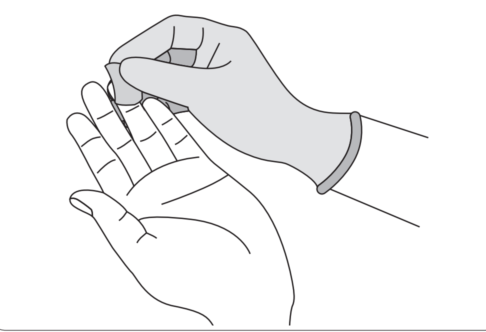

② Dry and pierce the wiped fingertip with a lancet to bleed.

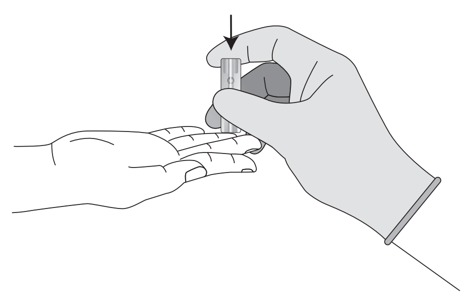

③ Take the inverted cup (1) provided and collect 5µl of the capillary whole blood specimen by dipping the circular end of the inverted cup into the specimen.

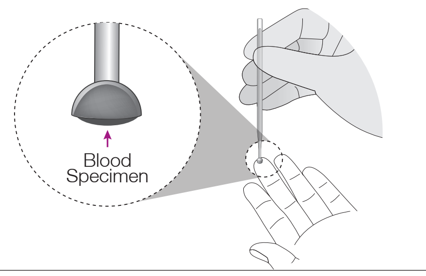

1-2. Collecting of Venous Whole Blood Specimen

Venous whole blood specimen can be collected into the commercially available anti-coagulant tube containing heparin, EDTA or sodium citrate by venipuncture.

2 **Applying of Specimen**

Apply the collected whole blood specimen to the sample well (circle) of the test device.

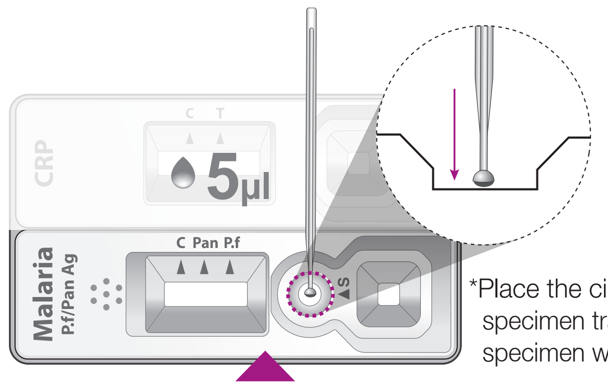

*Place the circular end of the specimen transfer device in the specimen well of the test device.

3 **Dropping of Assay Diluent**

Apply 3 drops (90µl) of assay diluent (2) to the assay diluent well (square) of the test device.

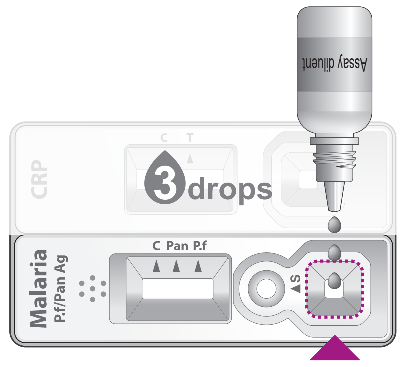

4 **Reading Time**

Read the test result after 15 minutes. The test can be read up to 30 minutes.

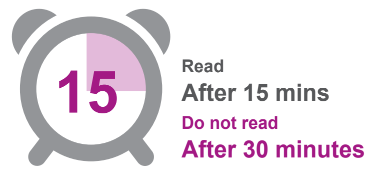

CAUTION Do not read test results after 30 minutes. It may give false results.

CRP test device

1

Use the STANDARD™ Ezi Tube+ (2) to collect the 10ul of whole blood specimen.

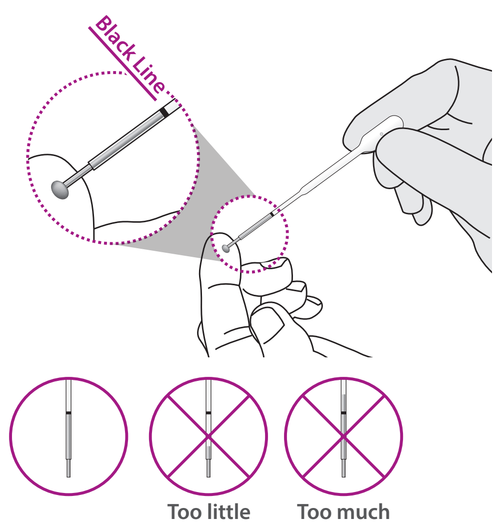

2

Add drawn specimen into an assay diluent (1).

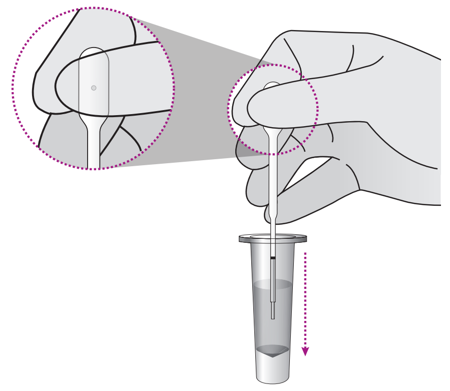

3

Discard used STANDARD™ Ezi Tube+ (2) in a sharps box.

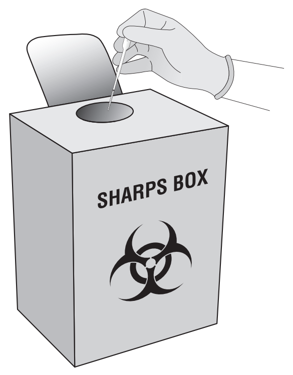

4

Use the disposable dropper (3) to mix the specimen and assay diluent. Carefully press and release a disposable dropper for 6-8times

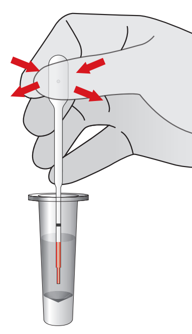

5

Collect ALL specimen using the disposable dropper (3)

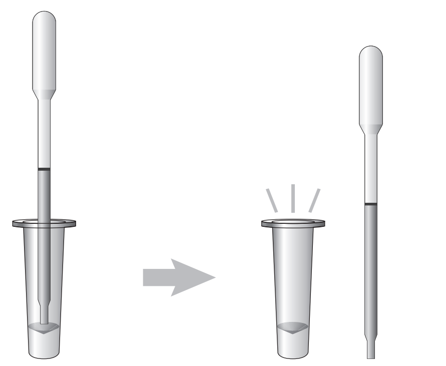

6

Add ALL specimen into the sample well of test device.

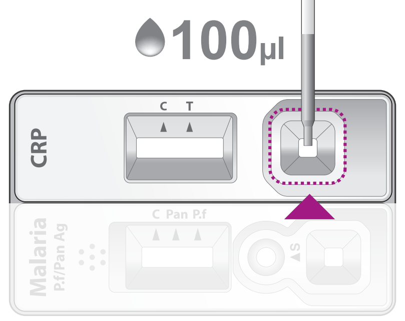

7

Interpret test results within 15-20 minutes after adding specimen.

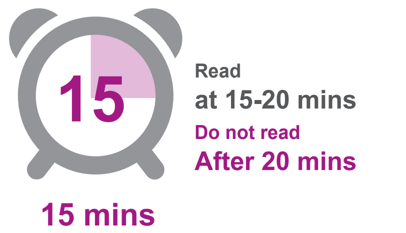

CAUTION Do not read test results after 20 minutes. It may give false results.

Interpretation of Test Results

Malaria P.f/Pan Ag test device

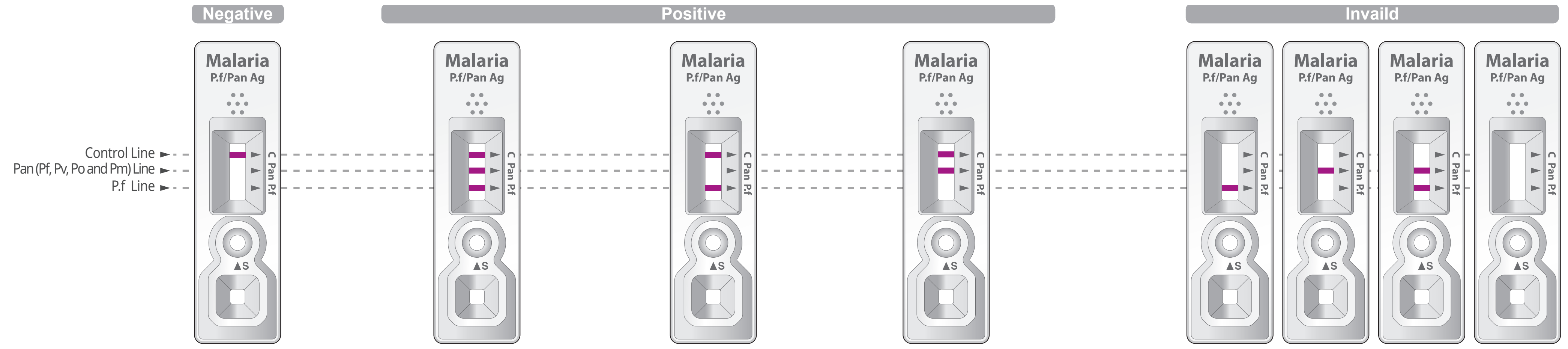

- **Negative:** The presence of only one colored band ("C" Control line) within the result window indicates a negative result.
- **Positive:** **Pf (*Plasmodium falciparum*) Positive or mixed Positive of Pf and Pv, Po, Pm**
- **Positive:** **Pf (*Plasmodium falciparum*) Positive**
- **Positive:** ***Plasmodium vivax, ovale* and/or *malariae* Positive or mixed positive of these**
- **Invalid:** If the control band ("C" Control line) is not visible within the result window, the result is considered invalid. The directions may not have been followed correctly or the test device may have deteriorated. Re-test with a new specimen and a new test device.

CRP test device

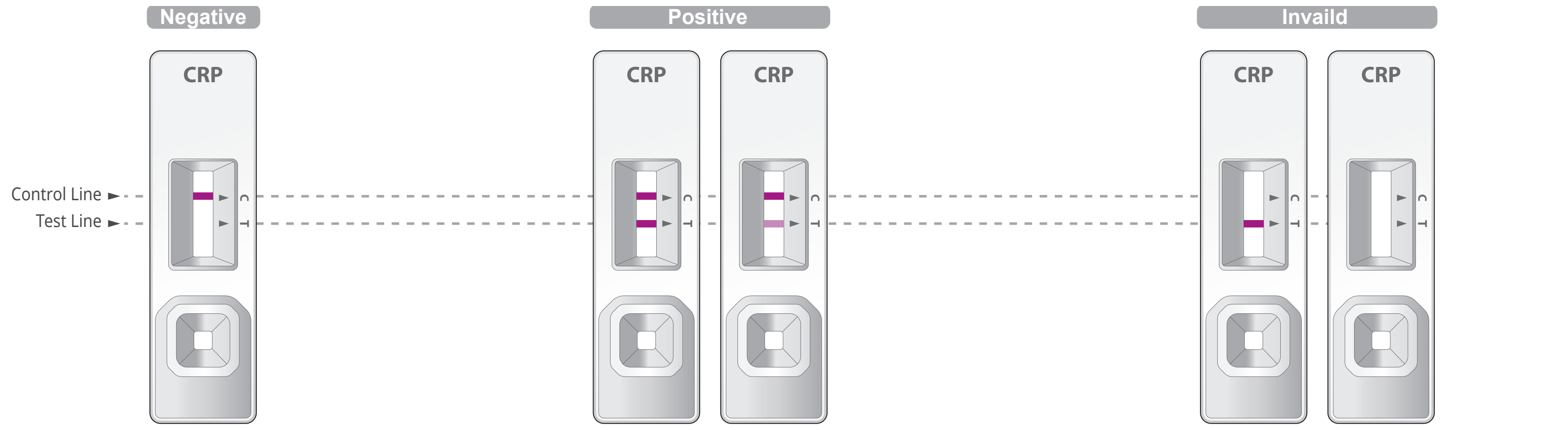

- **Negative:** CRP level is lower than 20mg/L
- **Positive:** CRP level is above 20mg/L
- **Invalid:** If the control band ("C" Control line) is not visible within the result window, the result is considered invalid. The directions may not have been followed correctly or the test device may have deteriorated. Re-test with a new specimen and a new test device.

EXPLANATION AND SUMMARY

[Introduction]

Malaria is caused by infection of red blood cells with protozoan parasites of the *Plasmodium* species. Into the human host by a feeding mosquito. The *Plasmodium* species trasnmitted from person to person are *P. falciparum, P.vivax, P.ovale* and *P.malariae*. First symptom of malaria are nonspecific and similar to viral illness. They comprise headache, fatigue, abdominal discomfort and muscle and joint pain, commonly followed by fever, chills, perspiration, anorexia, vomiting. At early stage of disease progression, with no evidence of vital organ dysfuction, a rapid, full recovery is expected, effective antimalarial treatment is given. If ineffective or poor-quality medicines are given or if treatment is delayed, particularly in *P.falciparum* malaria, the parasite burden often countries to increase and the patient may develop potentially lethal severe malaria. Accurate and prompt diagnosis of malaria is of utmost importance due to the morbidity associated with the other malarial forms. Rapid diagnostic test is an ideal diagnostic tool for malaria diagnosis in that it can provide a rapid determination if the patient is infected with malaria allowing for accurate treatment and improved outcomes.

CRP is a biomarker associated with bacterial infection. Tests that measure levels of CRP in the blood are routinely used to guide treatment decisions in high-income countries. Recent data suggest that CRP may also be suitable for use in Southeast Asia, a low malaria transmission setting. A combination malaria and CRP test that could be used at the point of care would give clinicians in low- and middle-income countries the ability to use a similar evidence-based approach to care, especially in low-burden malaria settings, where CRP has been shown to effectively differentiate bacterial from non-bacterial infections.

Over the past decade, the increased use of malaria RDTs has brought down the inappropriate use of antimalarial therapies by linking treatment to confirmed diagnosis. However, studies are now revealing that these tests may be inadvertently driving the over-use of antibiotics. Without a simple point-of-care test to determine whether an infection is bacterial, health providers may empirically prescribe antibiotics to patients with fever who test negative for malaria. In studies conducted in Malawi, Tanzania and Laos, patients with negative malaria RDT results had markedly higher odds of being unnecessarily prescribed antibiotics.

Antibiotics are not appropriate for viral or parasitic infections. Incorrect use of antibiotics contributes to the spread of antimicrobial resistance, endangers patients' health when serious illnesses are incorrectly treated and may increase health-care costs, which are often borne by patients out of pocket.

[Intended use]

STANDARD Q Malaria/CRP Duo Test is a rapid and membrane based immunochromatographic assay for malaria test device which qualitative detection of *Plasmodium falciparum (P. falciparum)* specific Histidine Rich Protein 2(HRP-2) and Plasmodium species (*P. falciparum, P. vivax, P. ovale* and *P. malariae*) specific Plasmodium lactate dehydrogenase (pLDH), for CRP(C-reactive protein) test device, as use to measure the CRP in the human whole blood specimen. This test is in vitro professional diagnostic use and intended as an aid to diagnosis of the infection in patient with suspected malaria before treatment is administered.

[Test principle]

STANDARD Q Malaria/CRP Duo Test contains two devices, the first device for Malaria P.f/Pan Ag, the second device for CRP test. Malaria P.f/Pan Ag test device contains three pre-coated lines, "P.f (*P. falciparum* line), "Pan" (*Plasmodium species: P. falciparum, vivax, ovale* and *malariae*) as test lines and "C" as control line on the surface of the nitrocellulose membrane. The test lines and control line in the result window of the test device are not visible before applying any specimens. Monoclonal anti-*P. falciparum* HRP-2 is coated on the P.f test line region, monoclonal anti-Malaria pLDH is coated on the Pan test line region and monoclonal anti-chicken IgY is coated on the control line region. During the test, the P. falciparum specific HRP-2 antigen and/or *Plasmodium* species specific pLDH in the specimen react to the gold-conjugated monoclonal anti-Malaria HRP-2 and/or gold-conjugated monoclonal anti-Malaria pLDH, and then bind to them respectively. Any *P. falciparum* specific HRP-2 antigen-antibody gold particle complex and/or *Plasmodium* species specific pLDH antigen antibody gold particle complex also migrate with the buffer and are immobilized by monoclonal anti-P. falciparum HRP-2 and/or monoclonal anti-Malaria pLDH at the two individual test lines to formation of violet test colored band(s) which confirms a positive result. Absence of this violet colored band indicates a negative result.The control line is used for procedural control, and should always appear if the test procedure is performed properly and the test reagents of the control line are working. CRP Test Device has pre-coated lines, "T" (CRP test line), and "C" (Control line) on the surface of the nitrocellulose membrane. For testing, the mixture of specimen and extraction buffer is added into the sample well. The immunoglobulin in the sample interacts with mAb anti-Human CRP that is coated on the test lines. If the CRP result is above the cut-off, the sample reacts with mAb anti-Human CRP conjugated to gold colloid. This produces a violet band in the test line, which confirms a positive result. Absence of this violet band in the test line indicates a negative result. The control line is used for procedural control, and should always appear if the test procedure is performed properly and the test reagents of the control line are working.

[Kit contents]

① Test device
② Assay diluent for Malaria P.f/Pan Ag
③ Inverted cup (5µl)
④ Lancet
⑤ Alcohol swab
⑥ Assay diluent for CRP
⑦ STANDARD™ Ezi tube+ (10µl)
⑧ Disposable dropper (100µl)
⑨ Instructions for use

[Materials required but not provided]

1. Anti-coagulant tube containing heparin, EDTA or sodium citrate for collection of venous whole blood
2. Venipuncture tools for venous whole blood collection
3. Timer
4. PPE (Personal Protective Equipment)

KIT STORAGE AND STABILITY

Store the kit at room temperature, 2-40°C / 36-104°F, out of direct sunlight. Kit materials are stable until the expiration date printed on the outer box. Do not freeze the kit.

WARNINGS

1. DO NOT FREEZE.
2. Do not use beyond the expiration date.
3. Do not re-use the test kit.
4. Do not use the test kit if the pouch is damaged or the seal is broken.
5. Do not use assay diluent of another lot.
6. Do not smoke, drink or eat while handling specimen.
7. Wear personal protective equipment, such as gloves and lab coats when handling kit reagents. Wash hands thoroughly afterwards.
8. Clean up spills thoroughly using an appropriate disinfectant.
9. Handle all specimens as if they contain infectious agents.
10. Observe established precautions against microbiological hazards throughout testing procedures.
11. Dispose of all specimens and materials used to perform the test as bio-hazard waste. Laboratory chemical and bio-hazard wastes must be handled and discarded in accordance with all local, state, and national regulations.
12. Silica gel in foil pouch is to absorb moisture and keep humidity from affecting products. If the moisture indicating silica gel beads change from yellow to green, the test device in the pouch should be discarded.
13. Carefully handling of lancet or needle while the specimen collection and preparation.

TEST PROCEDURE

[Preparation]

1. Carefully read the instruction for using the STANDARD Q Malaria/CRP Duo Test.
2. Look at the expiry date at the back of the foil pouch. Do not use the kit, if expiry date has passed.
3. Open the foil pouch, and check the test device and the silica gel pack within the foil pouch.

[Test procedure]

- **Malaria P.f/Pan Ag test device**

1. Clean a fingertip by wiping with an alcohol swab.
2. Dry and pierce the wiped fingertip with a lancet to bleed.
3. Take a inverted cup (5µl) provided and collect 5µl of the capillary whole blood specimen by dipping the circular end of the inverted cup into the specimen.
4. Venous whole blood specimen can be collected into the commercially available anti-coagulant tube containing heparin, EDTA or sodium citrate by venipuncture.
5. Apply the collected whole blood specimen to the sample well of the test device.
6. Apply 3 drops (90µl) of assay diluent into the assay diluent well of the test device.
7. Read the test result after 15 minutes. The test can be read up to 30 minutes.

- **CRP test device**

1. Clean a fingertip by wiping with an alcohol swab.
2. Dry and pierce the wiped fingertip with a lancet to bleed.
3. Use a STANDARD Ezi tube+ to collect the 10ul of whole blood specimen.
4. Venous whole blood specimen can be collected into the commercially available anti-coagulant tube containing heparin, EDTA or sodium citrate by venipuncture.
5. Add drawn specimen into an assay diluent.
6. Discard used STANDARD Ezi tube+ in a sharps box.
7. Use a disposable dropper to mix the specimen and assay diluent. Carefully press and release a disposable dropper for 6-8 times.
8. Collect all specimen using the disposable dropper.
9. Add all specimen into the sample well of test device.
10. Read the test result at 15-20 minutes. Do not read test result after 20 minutes. It may give false results.

INTERPRETATION OF TEST RESULT

[Malaria P.f/Pan Ag Test device]

1. Negative result: The presence of only one colored band ("C" Control line) within the result window indicates a negative result.
2. Pf (*P.falciparum*) Positive or mixed Pf (*P.falciparum*) and Pan (*P. falciparum, vivax, ovale* and *malariae*) positive result: The presence of three colored bands ("C" Control line ,"P.f" *P. falciparum* line and "Pan" *P. falciparum, vivax, ovale, malariae* line) within the result window, no matter which band appears first, indicates a Pf (*P.falciparum*) positive or mixed Pf (*P.falciparum*) and Pan (*P. falciparum, vivax, ovale* and *malariae*) positive result.
3. Pan (*P. falciparum, vivax, ovale, malariae*) positive result: The presence of two colored bands ("C" Control line and "Pan" *P. falciparum, vivax, ovale, malariae* line) within the result window, no matter which band appears first, indicates a Pan (*P. falciparum, vivax, ovale and malariae*) positive result.
4. Invalid result: If the control band ("C" Control line) is not visible within the result window, the result is considered invalid. The directions may not have been followed correctly or the test may have deteriorated. Re-test with a new patient specimen and a new test device.

[CRP Test device]

1. Negative result: The presence of only one colored band ("C" Control line) within the result window indicates that CRP level is lower than 20 mg/L.
2. Positive result: The presence of two colored bands ("C" Control line and "T" Test line) within the result window, no matter which band appears first, indicates that CRP level is above 20mg/L.
3. Invalid result: If the control band ("C" Control line) is not visible within the result window, the result is considered invalid. The directions may not have been followed correctly or the test may have deteriorated. Re-test with a new patient specimen and a new test device.

LIMITATION OF TEST

1. The test procedure, precautions and interpretation of results for this test must be followed strictly when testing.
2. STANDARD Q Malaria/CRP Duo Test is designed for human use.
3. Other clinically available tests are required if questionable results are obtained. As with all diagnostic tests, a definitive clinical diagnosis should not be based on the results of as single tests, but should only be made by the physician after all clinical and laboratory findings have been evaluated.

QUALITY CONTROL

1. A colored line appearing in the control line is an internal reagent and procedural control. It will appear if the test has been performed correctly and the reagent are reactive.
2. Control materials are not supplied with this test kit. However, it is recommended that the positive and negative controls be tested as a good laboratory practice to confirm the test procedure and to verify proper test performance.

BIBLIOGRAPHY

1. New rapid test could help bring down inappropriate use of antibiotics, FIND, March 16, 2017.
2. Technical Specifications Series submission to WHO Prequalification – Diagnostic Assessment. Malaria rapid diagnostics tests, TSS–3. Geneva: World Health Organization; 2017.
3. Paul, Rudrajit, et al. "Study of C reactive protein as a prognostic marker in malaria from Eastern India." Advanced biomedical research 1 (2012).
4. Imrie, Heather, et al. "Low prevalence of an acute phase response in asymptomatic children from a malaria-endemic area of Papua New Guinea." The American journal of tropical medicine and hygiene 76.2 (2007): 280-284.

CE IVD

Manufactured by **SD Biosensor, Inc.**
Head office : C-4th&5th, 16, Deogyeong-daero 1556beon-gil, Yeongtong-gu, Suwon-si, Gyeonggi-do, 16690, REPUBLIC OF KOREA
Manufacturing site : 74, Osongsaengmyeong 4-ro, Osong-eup, Heungdeok-gu, Cheongju-si, Chungcheongbuk-do, 28161, REPUBLIC OF KOREA

EC REP **Authorized Representative**
MT Promedt Consulting GmbH
Altenhofstrasse 80 66386 St. Ingbert Germany
Phone : +49 6894 581020, Fax : +49 6894 581021

Any inquiries regarding instructions provided should be addressed to: sales@sdbiosensor.com
or you can also contact us through www.sdbiosensor.com

L23MAL5ENR2
Issue date: 2019.09
