# Supplementary material for: Expanding the roles of community health workers to sustain programmes during malaria elimination: a meeting report on operational research in Southeast Asia
Source: Malar J. 2024 Jan 2;23:2. doi: 10.1186/s12936-023-04828-4 (PMC10759643; doi:10.1186/s12936-023-04828-4)
Supplement: Supplementary file 3 — Additional file 3. Organization of meeting discussions. [file 12936_2023_4828_MOESM3_ESM.pdf]

APPENDIX 3: Organization of meeting discussions

**August 2023**			
**Time**	**Activity**	**Speaker**	**Organization**
08:25 – 08:55	Registration		AHEAD
08:55 - 09:00	Welcome by Battambang PHD Director	Dr.Bunreth Voeurng	PHD
9:00 - 9:05	Welcome by MORU	Dr. Tom Peto	MORU
09:05 – 09:15	Opening remarks by CNM	HE, Prof. Lek Dysoley	CNM
**Session 1: New roles for village malaria workers**			
**Chair**	**Dr Bunreth Voeurng**		PHD
09:15 - 09:30	Why we need to sustain the VMW network	HE, Prof. Lek Dysoley	CNM
09:30 - 09:45	The RAI3E operational research overview	Dr. Moul Vanna	AHEAD
09:45 - 10:00	Health education packages	Mr. Sam Ol	AHEAD
10:00 - 10:15	SEACTN study results and planned trials	Dr. Tom Peto	MORU
**10:15 - 10:45**	**Break**		
**Session 2: New diagnostics for village malaria workers**			
**Chair**	**Dr Yok Sovann**		PHD
10:45 - 11:00	Dengue RDT deployment	Dr. Chan Davoeung	PHD
11:00 - 11:15	Malaria/CRP RDT deployment	Dr. Yok Sovann	PHD
11:15 - 11:30	Novel diagnostics at health centres	Dr. Tom Peto	MORU
11:30 - 11:45	G6PD biosensor use by VMWs	Dr. Bipin Adhikari	MORU

11:45 - 12:00	Summary of RAI3E operational research findings	Dr. Moul Vanna	AHEAD
**12:00 – 13:00**	**Lunch Break**		
**Session 3: The national and regional context of community health workers and malaria elimination**			
**Chair**	**Dr Chan Davoeung**		PHD
13:00 - 13:15	CNM's high-level strategy for the VMW network	HE, Prof. Lek Dysoley	CNM
13:15 – 13:30	Spot Sepsis results and disease severity biomarkers	Dr. James Callery	MORU
13:30 - 13:45	Malaria elimination strategies and community health workers – VMW roles	Céline Christiansen-Jucht (unable to attend)	WHO
13:45 - 14:00	Considerations for giving VMWs new roles?	HE, Prof. Lek Dysoley	CNM
**14:00 – 14:30**	**Break**		
**Session 4: Discussions**			
**Chair**	**Dr Dysoley Lek**		CNM
14:30 – 14:45	PHD perspectives on sustaining the VMW network	PHD Pailin & Battambang directors	PHD
14:45 - 15:00	Comments and closing remarks	HE, Prof. Lek Dysoley	CNM
